# Resident memory T cell development is gradual and shows AP-1 gene expression in mature cells

**DOI:** 10.1172/jci.insight.187381

**Published:** 2025-06-23

**Authors:** Neal P. Smith, Yu Yan, Youdong Pan, Jason B. Williams, Kasidet Manakongtreecheep, Shishir M. Pant, Jingxia Zhao, Tian Tian, Timothy Pan, Claire Stingley, Kevin Wu, Jiang Zhang, Alexander L. Kley, Peter K. Sorger, Alexandra-Chloé Villani, Thomas S. Kupper

**Affiliations:** 1 Center for Immunology and Inflammatory Diseases, Department of Medicine, Massachusetts General Hospital, Boston, Massachusetts, USA.; 2 Massachusetts General Hospital, Cancer Center, Boston, Massachusetts, USA.; 3 Broad Institute of Massachusetts Institute of Technology and Harvard, Cambridge, Massachusetts, USA.; 4 Department of Dermatology, Brigham and Women’s Hospital, Boston, Massachusetts, USA.; 5 Harvard Medical School, Boston, Massachusetts, USA.; 6 Ludwig Center at Harvard and Laboratory of Systems Pharmacology, Harvard Medical School, Boston, Massachusetts, USA.

**Keywords:** Immunology, Inflammation, Adaptive immunity, T cell development

## Abstract

Tissue-resident memory T (T_RM_) cells play a central role in immune responses across all barrier tissues after infection. However, the mechanisms that drive T_RM_ differentiation and priming for their recall effector function remains unclear. In this study, we leveraged newly generated and publicly available single-cell RNA-seq data generated across 10 developmental time points to define features of CD8^+^ T_RM_ across both skin and small-intestine intraepithelial lymphocytes (siIEL). We employed linear modeling to capture gene programs that increase their expression levels in T cells transitioning from an effector to a memory state. In addition to capturing tissue-specific gene programs, we defined a temporal T_RM_ signature across skin and siIEL that can distinguish T_RM_ from circulating T cell populations. This T_RM_ signature highlights biology that is missed in published signatures that compared bulk T_RM_ to naive or nontissue resident memory populations. This temporal T_RM_ signature included the AP-1 transcription factor family members *Fos, Fosb,* *Fosl2*, and *Junb*. ATAC-seq analysis detected AP-1–specific motifs at open chromatin sites in mature T_RM_. Cyclic immunofluorescence (CyCIF) tissue imaging detected nuclear colocalization of AP-1 members in resting CD8^+^ T_RM_ greater than 100 days after infection. Taken together, these results reveal a critical role of AP-1 transcription factor members in T_RM_ biology.

## Abstract 2

In this manuscript, we use temporal analyses to better understand resident memory T cells. We demonstrate the importance of AP-1 member transcription factors in Trm biology.

## Introduction

Adaptive immune memory mediated by T cells is central to host defense, and our appreciation of its complexity has evolved considerably over the last half century. Unanticipated heterogeneity in cytokine production by memory T cells was introduced in 1986, and migratory heterogeneity in circulating memory T cells was introduced in 1999 with the description of human central and effector memory T cells (T_CM_, T_EM_) (1, 2). Over the past 15 years, an additional population of resident memory T cells (T_RM_) has been the focus of many studies. T_RM_ cells have been shown to reside long-term in peripheral tissues, rather than circulating through blood or secondary lymphoid organs, and they play a critical role in antigen-specific recall immune responses (3).

To date, regulation of a series of key transcription factors has been associated with T_RM_ development and establishment. It has been well described that Hobit or its human analog Blimp1 are key regulators of T_RM_ maintenance that function via repression of genes associated with tissue egress (4). *Runx3* has been associated with T_RM_ establishment, specifically in CD8^+^ T_RM_, and is responsible for a cell’s responsiveness to the TGF-β signals associated with T_RM_ differentiation (5, 6). Conversely, T_RM_ precursors have been shown to lose expression of transcription factors that regulate tissue egress and lymph-homing molecules, including *Tcf7, Eomes* and *Klf2* (7, 8). More recently, a study utilizing single-cell RNA-seq (scRNA-seq) by Kurd et al. described how the expression of AP-1 transcription factor members *Junb* and *Fosl2* as well as *Nr4a2* appear to be essential for the development of T_RM_ in the small intestine, as their genetic deletion diminishes T_RM_ numbers (9).

CD8^+^ T_RM_ development and biology have been studied extensively in a number of infectious mouse models that employ transgenic T cells responsive to viral epitopes such as Vaccinia virus (VACV) and Herpes simplex virus (HSV) in skin as well as influenza in the lung (4, 10–17). Much of our additional knowledge on CD8^+^ T_RM_ in different tissues has emerged from similar mouse studies using lymphocytic choriomeningitis virus (LCMV) infection (6, 9, 18). Unlike VACV, HSV, and influenza, LCMV infection generates a systemic rather than a local immune response, leading to the formation of T_RM_ across many tissues, including gut, liver, lung, kidney, and salivary glands (19–24). While the local responses created by viruses such as VACV and the systemic responses induced by LCMV represent distinct biological phenomena, comparisons of the T cell differentiation processes in these 2 complimentary systems can help us to better define traits that are common in memory T cell development.

The present study leveraged scRNA-seq with MHC class I-restricted, ovalbumin-specific CD8 T cells (OT-Is) from the skin and draining lymph node (dLN) across a time course spanning 0–60 days after infection to study in more detail the evolution of T_RM_ and T_CM_ in parallel from the same infection. The granularity and cellular resolution provided by such scRNA-seq strategy performed across a time course enabled us to define previously uncharacterized heterogeneity among cells from the dLN in the early time points after infection. Additionally, our experimental approach utilizing a time course allowed for downstream linear modeling. This analysis captured gene programs associated with temporal T_RM_ development and defined genes associated with T_RM_ and T_CM_ cell fates in both our VACV-induced skin T cells, as well as publicly available LCMV-induced T cells in the small intestine. We identified progressively increasing AP-1 transcription factor family members as key genes contributing to our temporal T_RM_ signature across tissue compartments, which were confirmed to be associated with mature resting T_RM_ cells via ATAC-seq, CUT&RUN, and highly multiplexed, tissue-based CyCIF microscopy (25). In particular, the high expression and nuclear localization of AP-1 members in fully differentiated resting T_RM_ is unique among memory T cell lineages. These findings provide new insights into T_RM_ biology that could only be gained by an extensive temporal analysis and raise some potentially novel possibilities about how T_RM_ function in peripheral tissues.

## Results

### scRNA-seq of OT-I cells reveals 13 T cell subsets paving the way for memory T cell development across time and tissue sources.

**Figure 1:**
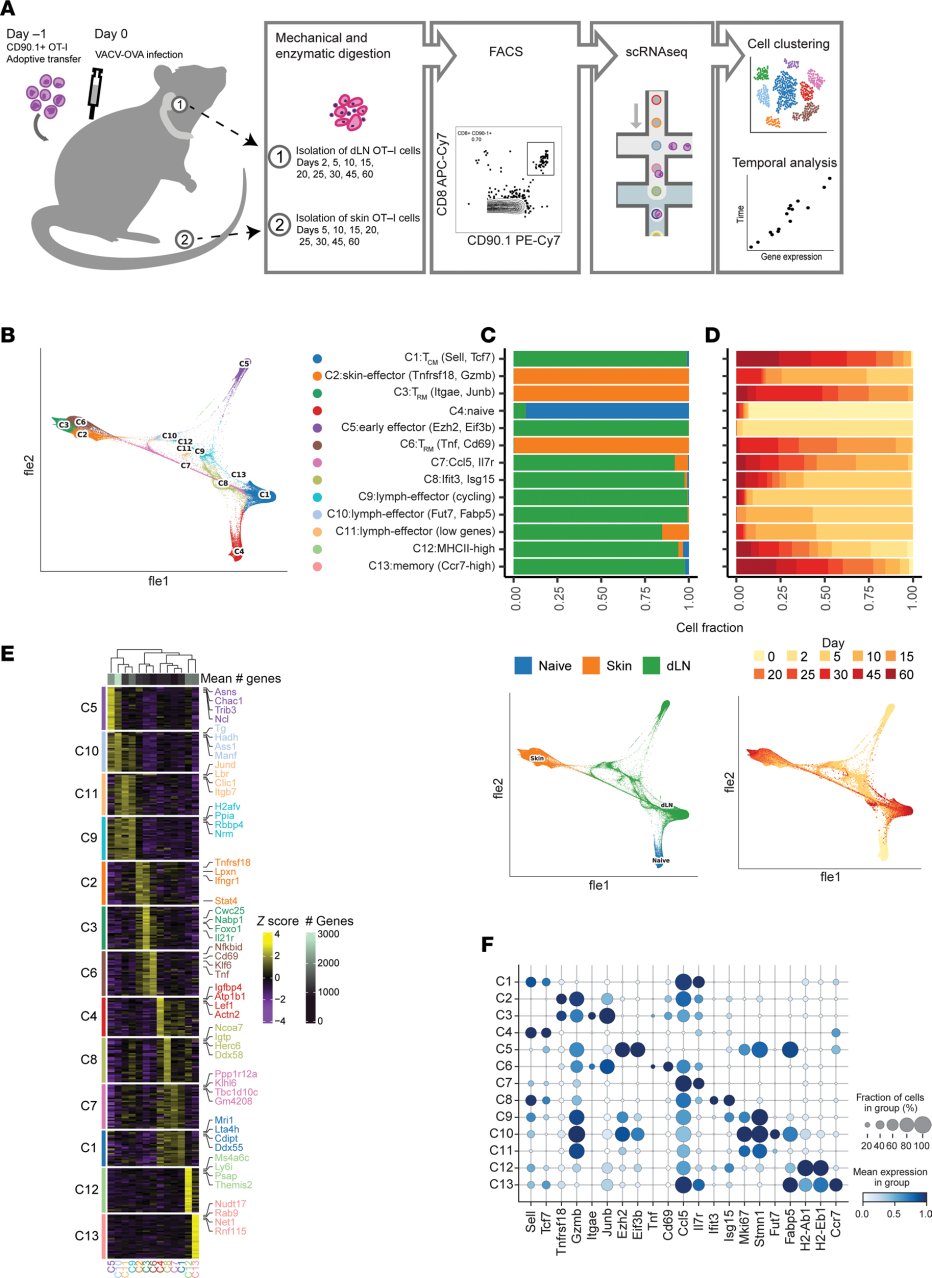
scRNA-seq of dLN and skin T cells in a viral infection model over time. (**A**) Schematic of the experimental design. (**B**) Force-directed layout embedding (FLE) of 63,265 high-quality single cells, colored by predicted Leiden cluster listed on the right. (**C** and **D**) (top) Source (**C**) and time point (**D**) composition of every cluster. Bars represent the fraction of cells in every cluster that were derived from the corresponding source or time point. (bottom) FLE embedding of cells pseudocolored by tissue source (**C**) or time point (**D**). (**E**) Heatmap showing the top discriminative gene sets for each cell cluster compared with every other cluster. Color scales denote the normalized gene expression (mean zero, unit variance) for each cluster and the mean number of genes captured per cluster (top bar). (**F**) Dot plot showing the percentage (size of the dot) and scaled expression (color) of known T-cell subset marker genes.

OT-I transgenic mouse T cells were adoptively transferred into recipient mice one day before skin infection with a recombinant VACV that expresses chicken ovalbumin peptide (amino acids 257–264) under the control of an early gene promoter (rVACV-OVA). Activated OT-I effector T cells were readily found in the skin as early as 5 days after infection and reached their maximum level at day 10 (data not shown), before beginning to decrease in number, as previously reported (17). scRNA-seq was performed on FACS-sorted OT-I cells from both skin and dLN, respectively, at serial time points from days 0–60 (Figure 1A and Supplemental Figure 1A; supplemental material available online with this article; https://doi.org/10.1172/jci.insight.187381DS1). After filtering out contaminating populations that lacked expression of canonical T cell markers (*Cd3d*, *Cd8a*, *Trac*), we recovered 63,265 high-quality cells across dLN and skin (Figure 1B).

When visualized in low-dimension space, our filtered dataset showed uniform expression of *Cd3d*, *Cd8a*, and *Trbc2*, with no contaminating non–T cell populations (Supplemental Figure 1B). To compare the data across both tissue sources and time point, we performed unbiased clustering analysis, which resulted in 13 distinct cell populations (Supplemental Figure 1C). The defined subsets were distinguished by different anatomical sites and time points, reflecting the emergence of different T cell subsets over time (Figure 1, B–D, and Supplemental Figure 1, D and E). To best visualize the trajectory of our cells, we used Force-directed layout embedding (FLE), a visualization tool designed to represent continuous developmental processes such as cell differentiation (26). Indeed, this algorithm has been used to successfully visualize temporal scRNA-seq data (27, 28). This dimensionality reduction approach grouped cells in order of time points measured—capturing the differentiation of naive T cells to effector and memory cells—while also maintaining distinctions created by tissue sources and Leiden clustering (Figure 1, B–D). All subsets were defined by a distinct set of genes using statistically complementary strategies (AUC ≥ 0.75, one-vs-all [OVA] pseudobulk FDR < 0.05), justifying our cluster resolution (Figure 1E and Supplemental Table 1). C4 was almost exclusively comprised of naive T cells and showed expression of lymph-homing markers *Sell* and *Ccr7* (Figure 1F). We identified a subset of T_CM_ cells (C1) that shared many phenotypic markers with naive cells but came from late time points (Figure 1D), likely reflecting the similar quiescent state of naive and T_CM_ cells. There was an additional subset of dLN memory cells with high *Ccr7* expression (C13), but they represented a very small proportion of the captured cells (133 cells, 0.21%) (Supplemental Figure 1F).

Five dLN populations (Figure 1C and Supplemental Figure 1D) were identified as having an effector-like phenotype that broadly expressed markers of cytotoxicity (e.g., *Gzma*, *Gzmb*) (Figure 1F). These populations included C5 early effector cells that were largely made up by day 2 cells and were defined by markers known to be associated with early antigen activation such as *Ezh2* and *Eif3b* (29, 30). Additional populations of dLN effector T cells included the C8 population expressing high levels of IFN stimulated gene (ISG) signature (e.g., *Ifit3*, *Isg15*), the C9 population defined by high levels of cell cycle markers, the C10 population marked by the expression of the known skin-homing marker *Fut7* and T_RM_ -associated gene *Fabp5* and the C11 population that seemed to be defined by having a lower number of genes detected, possibly reflecting lower-quality cells (Figure 1, E and F, and Supplemental Figure 1G). Over 75% of cells from C8–11 populations were from day 10 or earlier (Figure 1D). A small population of dLN cells defined by high expression of MHC-II machinery (C12) was also present in the data (*n* = 449 cells, 0.71%) (Supplemental Figure 1F). Given their small representation and our inability to distinguish doublets, this population was not explored further.

In the skin, three distinct populations were defined (Figure 1, B and C, and Supplemental Figure 1D). C2 represented the earliest of the skin T cells with greater than 50% of cells from days 5 and 10 (Figure 1D and Supplemental Figure 1F), with an effector T cell phenotype defined by *Tnfrsf18*, *Gzmb*, and *Ctla4* expression (Figure 1E and Supplemental Table 1). In contrast, C3 and C6 were defined as T_RM_ cells because they were made up largely from the later time points (greater than day 15) and nearly absent in the earliest time points (before or on day 10). C3 expressed the highest level of *Itgae* and *Icos*, while C6 expressed higher levels of *Cd69* and *Tnf*. Interestingly, C3 and C6 were represented in nearly equal proportions in the middle time points (days 15–25: C3 = 43.7% of skin cells, C6 = 44.0% of skin cells) (Supplemental Figure 1F). However, C3 became the dominant T_RM_ population by the end of the time course (days 45–60: C3 = 87.2% of skin cells, C6 = 12.2% of skin cells), suggesting that C3 represented the mature resting T_RM_ population.

### T cell subsets with skin-homing features defined in lymph node.

**Figure 2:**
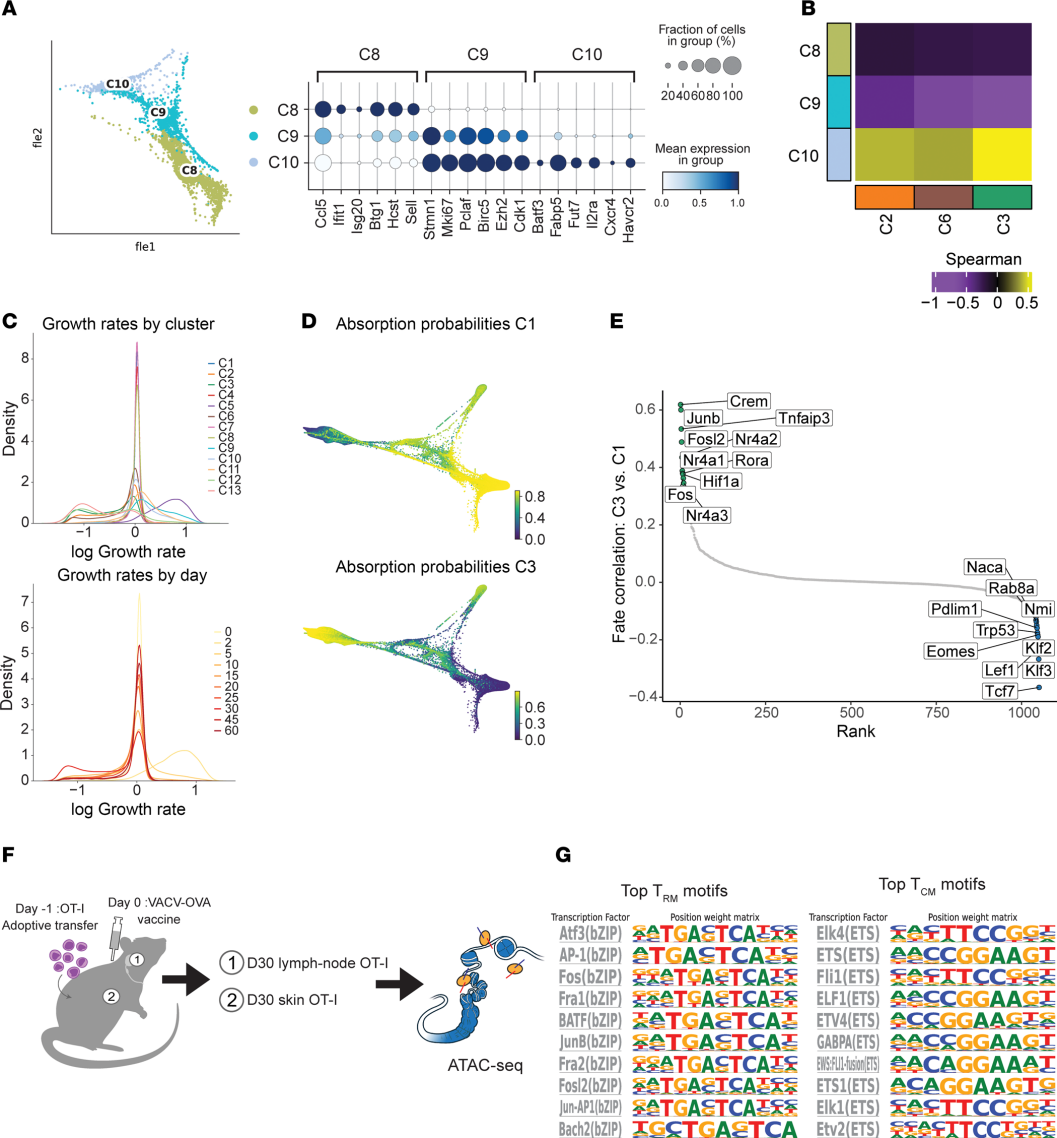
Heterogeneity of antigen-specific T cells early postinfection and transcription factors the drive memory T cell differentiation. (**A**) (left) FLE of clusters most associated with day 5 dLN cells (C8, C9, and C10). (right) Dot plot showing the percentage (size of the dot) and scaled expression (color) of select marker genes for each of the 3 clusters. (**B**) Pairwise Spearman correlation between the OVA log_2_ fold-change values of clusters C8, C9, and C10 versus C2, C6, and C3. (**C**) A growth rate was calculated by comparing the relative expression of genes involved in proliferation versus apoptosis. Histograms show distribution of this growth rate across all cells when grouped by cluster (upper panel) or grouped by time point (lower panel). (**D**) Probabilities of cells reaching the C1 (top) or C3 macrostate (bottom) as determined by absorption probabilities. Color scale represents probability of a cell to reach the given cell state (blue, low probability; yellow, high probability). (**E**) Transcription factors most associated with each Waddington-OT determined mature cell state. The top 10 transcription factors associated with C3 state (left, top) and C1 state (right, bottom) are labeled. (**F**) Schematic of the ATAC-seq experimental design. (**G**) HOMER-known motif analysis comparing T_RM_ and T_CM_ samples profiled. Shown are the transcription factors and position weight matrices for the top 10 known motifs for T_RM_ (left) and T_CM_ (right).

We sought to assess in which tissue compartment (dLN versus skin) and at which time point T cell subset differentiation start emerging through the course of T cell development. Our first 2 time points revealed little heterogeneity, with 96% of naive day 0 cells being found in C4 and 98% of day 2 cells belonging to C5 (Supplemental Figure 1F). Interestingly, cell subset diversification was first observed at day 5 across three distinct clusters (C8, C9, and C10) representing 85% of day 5 cells from the dLN (Supplemental Figure 1F and Supplemental Figure 2A). C8 was enriched in IFN-response genes (*Ifit1*, *Isg20*) as well as *Btg1*, a gene associated with T cell quiescence (31) (Figure 2A and Supplemental Figure 2B). Additionally, C8 had the highest expression of the lymph-homing molecule *Sell*. Subsets C9 and C10 were enriched for cell cycle markers (*Stmn1*, *Mki67*, *Pclaf*, *Birc5*), while only C10 expressed genes associated with skin-homing and T_RM_ development (*Fabp5*, *Fut7*). Interestingly, the presence of C8 and C9 cells persist through our time course (21% of C8 were from later than day 15 and 3.7% of C9 cells were from later than day 15 cells), while C10 is almost completely absent by day 15 (0.1% of C10 cells were from later than day 15), suggesting C10 is a transient cell state in the dLN (Supplemental Figure 1F). To understand the relationship between these dLN populations (C8, C9, and C10) and the defined skin cell subsets (C2, C6, and C3), we used each cluster’s gene expression profile to calculate pairwise Spearman correlations (Figure 2B), which showed that only the expression profile of C10 correlated with C2, C6, and C3. These results support the hypothesis that the emergence of cell subset diversification among antigen-activated T cells appears before trafficking to peripheral tissues.

### AP-1–family transcription factors are associated with T_RM_ development.

We next employed the Waddington-Optimal Transport (Waddington-OT) algorithm through the CellRank suite of tools (28, 32). Waddington-OT infers temporal couplings between cells profiled across our experimental time course, capturing the transcriptional programs and regulators driving the transition between cell states (28). To determine if the predicted rate of cellular proliferation should be considered when modeling cellular trajectories, we calculated a growth rate based on expression of genes associated with the cell cycle and apoptosis (Figure 2C). The predicted growth rates across the 13 clusters and 10 time points were uniform, with the exception of C5, which was largely made up of day 2 cells and most associated with a higher growth rate (mean log growth rate of C5: 0.64, mean log growth rate of all other clusters: –0.13). However, given that this cluster represented only a small fraction of our dataset (7,130 cells, 11.3%), we opted for modeling the data with a uniform growth rate.

When using the Waddington-OT algorithm to predict 2 mature T cell macrostates (regions of the phenotypic manifold that cells are unlikely to leave), the subsets identified as the most likely to represent mature differentiated cells were C1 and C3, which correspond to late time point dLN (T_CM_) and skin cells (T_RM_), respectively (Figure 2D). To elucidate drivers of memory T cell differentiation, we looked at the transcription factors most associated with either the C1 or C3 lineages (Figure 2E and Supplemental Table 2). While the C1 trajectory was associated with transcription factors known to be associated with lymph homing and T cell memory (*Tcf7, Eomes*) (33), the C3 trajectory strongly correlated with AP-1 transcription factor members *Junb*, *Fosl2*, and *Fos*, along with other additional known immediate early genes *Nr4a1*, *Nr4a2*, and *Nr4a3*.

To investigate further the transcriptional regulators driving memory T cell differentiation, we performed ATAC-seq on D30 post-VACV skin infection T cells, including skin T_RM_ and dLN T_CM_ cells (Figure 2F, Supplemental Figure 1A, and Supplemental Figure 2C). We probed for differences in transcription factor motifs between these 2 T cell subsets using HOMER motif analysis and, consistent with our transcriptional signatures, we found the T_RM_ cells to be strongly enriched with bZIP family transcription factor motifs including AP-1, *Fos*, *JunB*, and *Fosl2*, validating the regulators predicted by the Waddington-OT algorithm. Conversely, T_CM_ were enriched for ETS family transcription factor motifs, including those corresponding to *Ets1*, *Elk1*, and *Elk4* (Figure 2G and Supplemental Table 3).

### Gene programs leading to a resident-memory state in Skin and siIEL T cells include distinct features.

**Figure 3:**
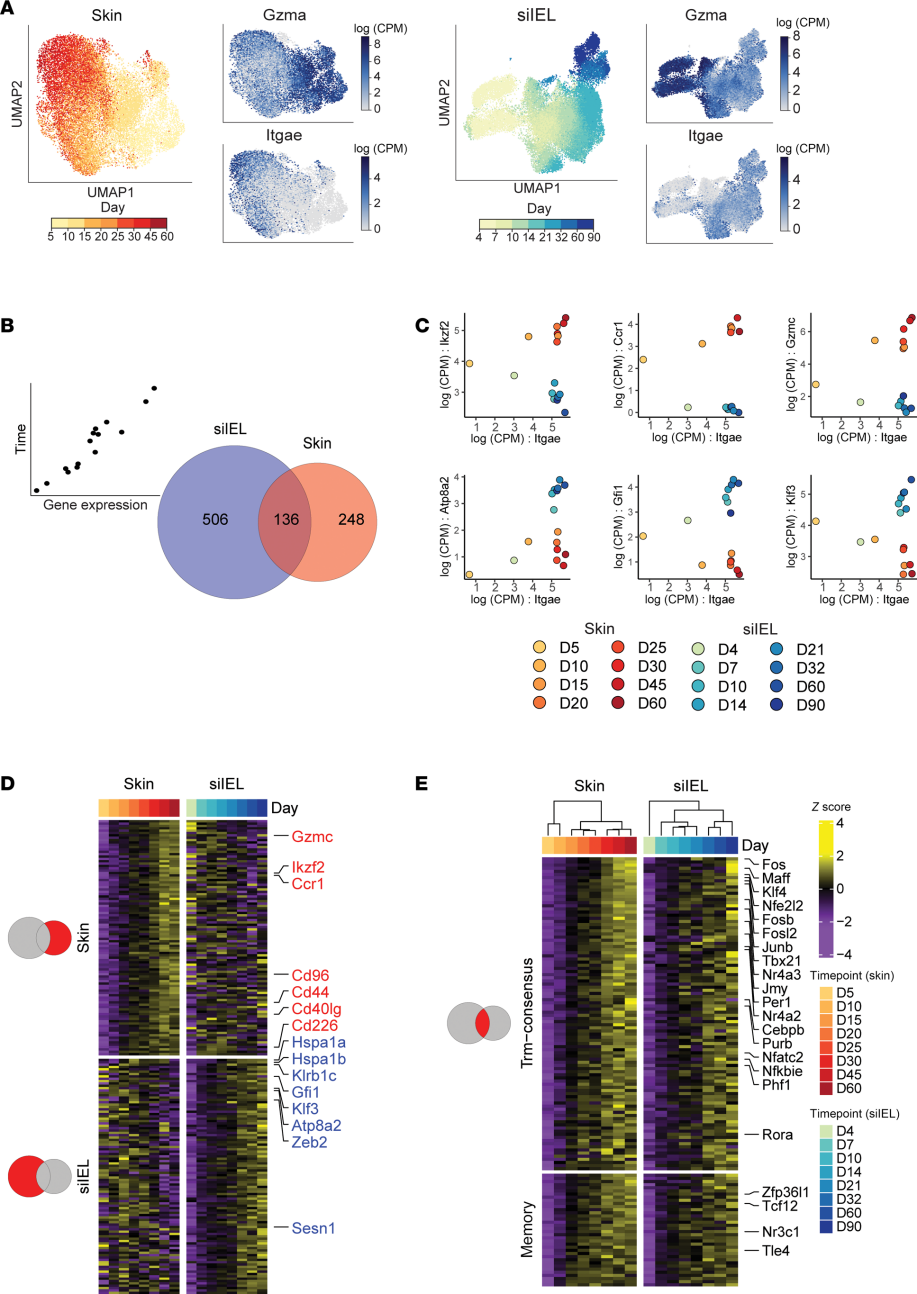
Linear modeling reveals tissue-specific and consensus temporal T_RM_ gene signature in viral infection models. (**A**) Uniform Manifold Approximation and Projection (UMAP) embedding of skin (left) and siIEL scRNA-seq data (right) pseudocolored by experimental time point. To the right of each time point UMAP are feature plots using color to indicate gene expression levels (Log(CPM)) of *Gzma* and *Itgae*. (**B**) Venn diagram of the significant T_RM_-associated genes in siIEL (left) and skin (right) as determined by linear modeling. (**C**) Scatter plots showing the Log(CPM) of select skin-specific (top) and siIEL-specific (bottom) T_RM_ genes on the *y* axis and Log(CPM) of *Itgae* on the *x* axis. Color scale indicates both anatomical location and time point the sample was from. (**D**) Heatmap showing the top 100 genes unique to the skin (top) and siIEL (bottom) T_RM_ signatures. Top bar indicates the associated timepoints. Color scales denote the normalized gene expression (mean zero, unit variance) for each timepoint. (**E**) Heatmap showing the temporal T_RM_ gene signature across timepoints in both skin (left) and siIEL (right) datasets. Color scale denotes normalized gene expression (mean zero, unit variance) for each timepoint. The genes on top represent those unique to the T_RM_ signature genes (*n* = 100), while the genes on the bottom represent those additionally found in the T_CIRC_ signature (*n* = 36). Transcription factors are labeled on the right.

To better understand genes that are associated with T_RM_ differentiation, skin T cells were subclustered independently for further downstream analysis. Additionally, we included in our analysis a previously published dataset that used similar time course kinetics to analyze the differentiation of siIEL T_RM_ cells using a LCMV infection model (9) (Figure 3A). We sought to examine the shared and distinct dynamics of transcriptional changes across the T cell differentiation spectrum, which traditionally have been thought to be phasic going from a naive to effector to memory state. We first looked at the top 500 genes by variance in each dataset across time points, which revealed that the differentiation into a memory T cell state was not phasic but gradual in the weeks after infection. The dominant direction of transcriptional changes in both the skin and siIEL were found to be either positive (skin, 261 genes; siIEL, 241 genes) or negative (skin, 203 genes; siIEL, 221 genes) linear correlations with time, while a much smaller fraction of genes were found to be expressed highest in the middle timepoints (skin, 36 genes; siIEL, 38 genes) (Supplemental Figure 3A). To identify genes associated with T_RM_ development, a linear model was fit to gene expression data, capturing genes that gradually increase from the early time points to later time points in each tissue. This approach defined 642 and 384 T_RM_ genes in the siIEL and skin respectively (FDR < 0.1, regression slope > 0.15; Figure 3B and Supplemental Table 4). The majority of T_RM_ genes were unique to a single anatomical site (siIEL, 506 unique genes; skin, 248 unique genes). For example, the transcription factor *Ikzf2* (Helios; *P* value = 5 ×10^–4^) was specific to skin (Figure 3, C and D). In addition to correlating with time, we can see that *Ikzf2* also correlates with the expression of the canonical T_RM_ gene *Itgae* in skin, further supporting the notion that this gene is important for T_RM_ formation. There were additional immune mediators specific to skin, including *Ccr1* (*P* value = 0.001) and *Gzmc* (*P* value = 0.0001). In the siIEL compartment, there was an enrichment of heat-shock proteins (HSPs) associated with T_RM_ development (*Hspa1a*, *Hspa1b*, *Hsph1*, *Hsp90aa1*, *Dnaja4*) which have not been described in this context. Other siIEL T_RM_–defining transcripts included *Atp8a2*, (*P* value = 0.002) a gene recently reported to be associated with siIEL regulation as well as transcription factors *Klf3* (*P* value = 2 ×10^–6^), *Tox* (*P* value = 0.0001) and *Gfi1* (*P* value = 0.003) (34). The expression of these genes also correlated with *Itgae* in the siIEL, but not skin, further supporting their tissue specificity (Figure 3C). GSEA was then performed using both the HALLMARK and KEGG databases to identify gene sets that are associated with T_RM_ development. Skin T_RM_ development was distinctively associated with apoptosis and IL2 signaling pathways, while gene sets associated with siIEL T_RM_ development included those for Wnt and Notch signaling (Supplemental Figure 3, B and C, and Supplemental Table 5). This analysis also revealed 13 shared T_RM_ gene sets, including the Hallmark Hypoxia (skin NES = 1.90, *P* value = 0.0001; siIEL NES = 1.47, *P* value = 0.007) and TGF-β signaling (skin NES = 2.19, *P* value = 0.0001; siIEL NES = 1.80, *P* value = 0.001) pathways (Supplemental Figure 3D and Supplemental Table 5), suggesting shared core programs leading to resident-memory states in skin and siIEL T cells. In addition, there were 27 shared down-regulated T_RM_ gene sets, including those associated with DNA replication and cell cycle progression, which would be expected of T cells entering a quiescent state.

### dLN and spleen T cells from VACV and LCMV models share transcriptional programs that lead to circulating memory T cell development.

To better understand the development of circulating memory T cells (T_CIRC_) across different viral infection systems, we employed the same linear modeling approach with dLN cells from our VACV infection time course and spleen cells from the LCMV infection time course dataset (9) (Supplemental Figure 4, A and B). This approach defined 492 and 1212 dLN- and spleen-T_CIRC_ genes respectively (FDR < 0.1, regression slope > 0.15; Supplemental Table 4). Unlike the distinct gene profiles observed between skin and siIEL T_RM_ cells, the majority of dLN T_CIRC_ genes from the VACV model were shared with spleen T_CIRC_ genes from the LCMV model (*n* = 396 genes, 80%) (Supplemental Figure 4B). Notably, the majority the dLN-specific genes in the spleen time course were most highly expressed at the latest time points (Supplemental Figure 4C). This would suggest that, while strict statistical criteria did not allow them to be considered T_CIRC_ genes in the spleen, many may still be involved in T_CIRC_ differentiation in dLN and spleen. In contrast, several T_CIRC_-specific genes found in the spleen of LCMV-infected mice were not upregulated in the dLN T_CIRC_ of VACV-infected mice (*n* = 816 genes) (Supplemental Figure 4B). This could plausibly be attributed to distinct cellular compositions of the T_CIRC_ populations at each anatomical site. While the dLN will predominantly contain T_CM_ cells, the spleen comprises a mix of T_CM_ and T_EM_.

### Skin and siIEL T cells from VACV and LCMV models share core transcriptional programs essential to resident memory T cell development.

**Figure 4:**
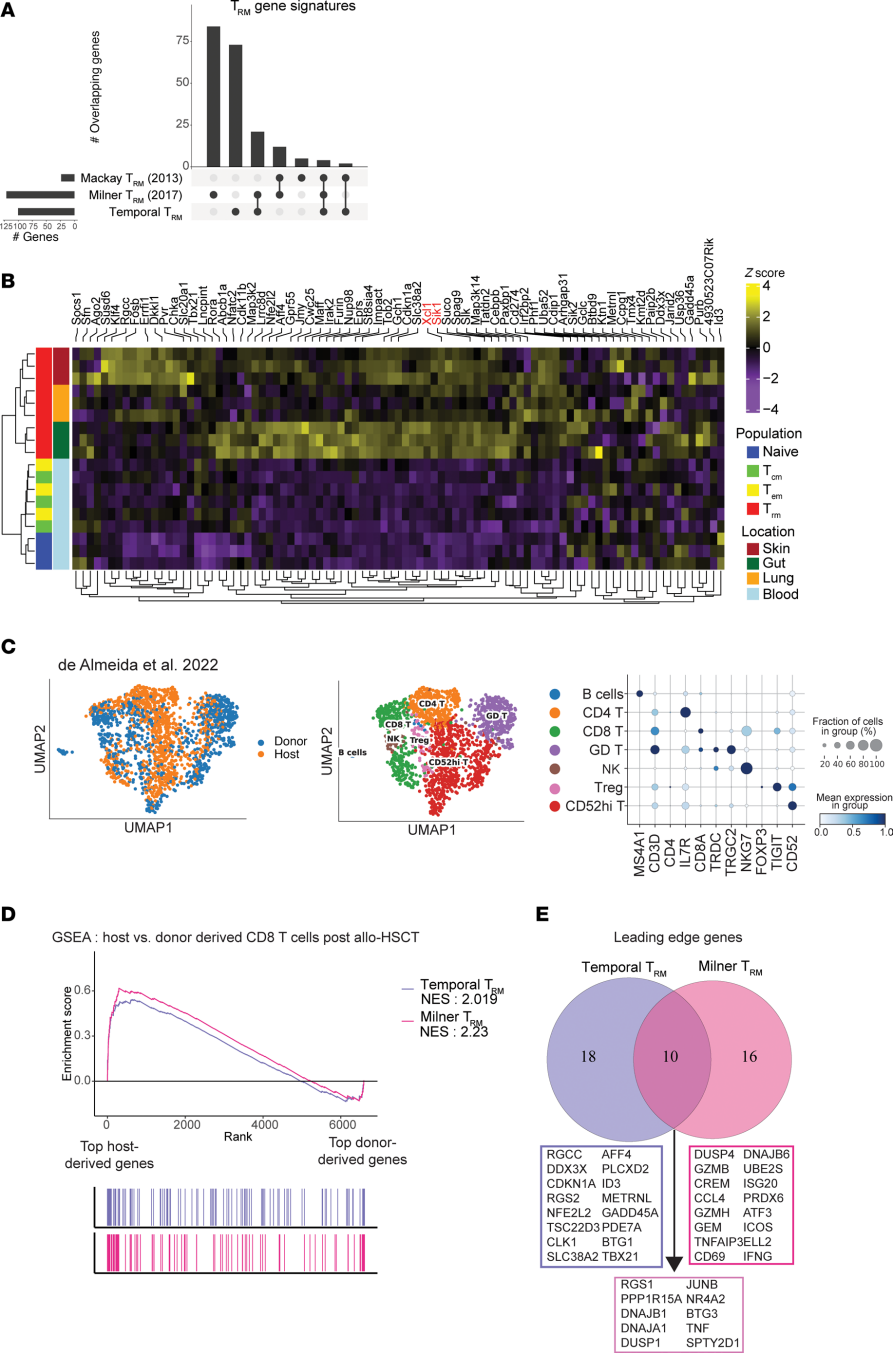
Temporal T_RM_ gene signature distinguishes T_RM_ in mouse and human models. (**A**) UpSet plot showing the overlap between our temporal T_RM_ signature and two previously published signatures by Milner et al. (6) and Mackay et al. (16). Each column represents a unique intersection, as shown by the dark points in the dot-matrix. Bars for each column represent the size of the overlap between each combination. Bars on left represent the size of each unique T_RM_ gene set. (**B**) Heatmap showing expression levels of our temporal T_RM_ gene signature across T cell subset microarray samples publicly available from Mackay et al. (20). Color scales denote the normalized gene expression (mean zero, unit variance) for each sample. Genes listed in black are unique to our temporal T_RM_ signature. Genes listed in red are those that are shared among all 3 T_RM_ signatures. (**C**) UMAP embedding of 1,829 skin lymphocytes from a donor 796 days after allogenic hematopoietic stem cell transplantation (42) colored by T cell source (left) and annotated cell type (middle). (right) Dot plot showing the percentage (size of the dot) and scaled expression (color) of select marker genes for the annotated cell types. (**D**) Host versus donor-derived CD8^+^ T cells were compared and genes associated with each were ranked (highest rank = genes associated with host-derived CD8^+^ T cells, lowest rank = genes associated with donor-derived T cells). This ranking was used as input to GSEA using the temporal T_RM_ gene set and the T_RM_ gene set published by Milner et al. (6). (**E**) Venn diagram of the leading edge genes associated with the GSEA analysis shown in **D**.

While many of the defined T_RM_ genes were distinct in our 2 anatomical niches, we found a consensus T_RM_ signature of 136 genes that were commonly expressed among both skin and siIEL compartments (Figure 3, B and E, and Supplemental Table 4; FDR < 0.1, regression slope > 0.15). Of these, 100 genes were unique to T_RM_ and 36 genes were also included in our T_CIRC_ signature. We predict that these genes are important for general T cell memory and include *Btg2* and *Bcl2,* factors known to mediate T cell quiescence and memory (Supplemental Table 4) (31, 35). When considering the expression of our temporal T_RM_ signature across our defined cell clusters, we see the highest expression in C3 (Supplemental Figure 5A). Previous studies generated gene sets associated with T_RM_ development by comparing fully differentiated T_RM_ to other differentiated memory T cell subsets (T_CM_, T_EM_). To highlight the strength of our temporal linear modeling to derive a T_RM_-related gene signature, we compared our results to two previously published T_RM_ gene signatures (6, 16) (Figure 4A). Of the 100 genes found in our temporal T_RM_ signature, 27 were found in at least one of the other gene sets, with 3 genes being found across all gene signatures (*Xcl1*, *Sik1*, *Rgs1*). 73 genes were unique to our approach, including *Id3,* a transcription factor that has been associated with T cell memory (36) and the immediate early genes *Fosb* and *Cebpb*. Importantly, hierarchical clustering of naive, T_CM_, T_EM_, and T_RM_ microarray samples (16) based on the 100 gene temporal T_RM_ signature perfectly segregated T_RM_ from the others, further validating the use of linear modeling to capture genes defining T_RM_ (Figure 4B). Noteworthy, when we examine the genes unique to the largest current T_RM_ gene set (6) (*n* = 121 genes) that was generated with microarray data, their expression patterns do not consistently track with T_RM_ development in the skin and siIEL (Supplemental Figure 5B). In addition to the different analytical approaches taken to generate our temporal T_RM_ gene set versus the previously published gene sets, many discrepancies may be due to the differences in technologies used to examine gene expression, as certain genes are not well captured by scRNA-seq.

To understand whether the temporal T_RM_ gene signature we defined in mice were also found associated with human T_RM_, we reanalyzed a publicly available dataset from De Almeida et al. that examined T_RM_ in the context of tissue transplantation. This study performed scRNA-seq on immune cells isolated from the skin of a patient 796 days after allogenic hematopoietic stem cell transplantation (allogenic-HSCT) (37) and included cells that were bona fide T_RM_, which were identified by the presence of recipient single-nucleotide variants (SNVs), as well as donor T cells who lacked these SNVs and are predicted to be a mix of resident and nonresident T cells (Figure 4C). Our reanalysis of this dataset was able to identify CD4^+^ T cells, CD8^+^ T cells, γ-δ T cells, NK cells, regulatory T cells (Tregs), CD52-high T cells, and B cells from the transplant donor and recipient. When performing GSEA on the CD8^+^ T cells, we found that our temporal T_RM_ gene set was associated with the recipient-derived T_RM_ when compared to the donor-derived cells (Figure 4D). Notably, a similar pattern was seen when using the largest current T_RM_ gene set from Milner et al. (6). However, the signal from the temporal T_RM_ signature was driven by unique genes not found in other gene sets, including *RGS2*, *ID3*, and *BTG1* (Figure 4E).

As further validation, we analyzed the T cells from the skin of 4 healthy donors published by Chennareddy et al. to see if we could find a subset of cells that had a gene expression pattern consistent with our temporal T_RM_ signature (38) (Supplemental Figure 5C). Reclustering this data identified 3 distinct subsets of CD8^+^ T cells, γ-δ T cells, NK cells and Tregs (Supplemental Figure 5D). When performing GSEA, two CD8^+^ T cell clusters (chenn_CD8T_1, chenn_CD8T_3) were significantly associated with our temporal T_RM_ gene set as well as the T_RM_ gene set from Milner et al., including a population defined by the AP-1 members *JUNB* and *FOS* (Supplemental Figure 5, E and F). Similar to our analysis of the allogenic-HSCT data, the signal from each gene set was driven by genes not found in the other gene set (Supplemental Figure 5G). We then set out to analyze public scRNA-seq data from Boland et al. that included T cells from the rectum and peripheral blood mononuclear cells (PBMCs) of 9 healthy individuals (Supplemental Figure 5H) (39). Reclustering this data identified 3 CD4^+^ T cell populations, 2 CD8^+^ T cell populations, and Tregs (Supplemental Figure 5I). Three populations (boland_CD4T_1, boland_CD8T_1, boland_Treg) were enriched in the rectum when compared with PBMCs (Supplemental Figure 5J). Importantly, the CD8^+^ cluster enriched in the rectum was significantly associated with both the temporal T_RM_ gene set as well as the T_RM_ gene set from Milner et al., with many genes driving the signal in the 2 gene sets being distinct (Supplemental Figure 5, K–M). Taken together with the allogenic-HSCT model of T_RM_ in humans, these analyses demonstrated the additive nature of a linear model approach to finding genes associated with memory T cell development.

Previous work has demonstrated that the regulation of key transcription factors are important for T_RM_ development, such as *Runx3*, *Notch1*, and *Zfp683* (4–6, 40). When looking at our temporal T_RM_ signature, 20 of the genes identified were transcription factors. These included AP-1 family members (*Fos*, *Fosb*, *Fosl2*, *Junb*, *Maff*), 2 members of the NR4A family (*Nr4a2*, *Nr4a3*) as well as *Rora* (Figure 3E). In addition to the results of our linear modeling on gene expression data, we defined T_RM_-associated transcription factors that are both tissue-specific and shared across the skin and siIEL compartments using a modified version of the SCENIC algorithm. This algorithm takes into account both expression of transcription factor genes and those with the potential to be targeted by a given transcription factor based on the presence of specific motifs, creating “AUCell” scores, which could then be examined over time (Supplemental Figure 6A and Supplemental Table 6) (41, 42). The skin-specific regulons identified (FDR < 0.1) included *Klf6* (*P* value = 8.5 × 10^–5^) *Irf4* (*P* value = 0.004), *Nfil3* (*P* value = 1.2 × 10^–5^), and *Spi1* (*P* value = 0.001). Several siIEL-specific regulons were also identified (FDR < 0.1) that included *Foxo3* (*P* value = 0.001), *Elf2* (*P* value = 0.01), and *Myc* (*P* value = 0.04), which have all been reported to either inhibit proliferation and/or have been associated with T_RM_ development in an LCMV model (9, 43, 44).

### AP-1 transcription factor family members are common to T_RM_ across different anatomical niches.

**Figure 5:**
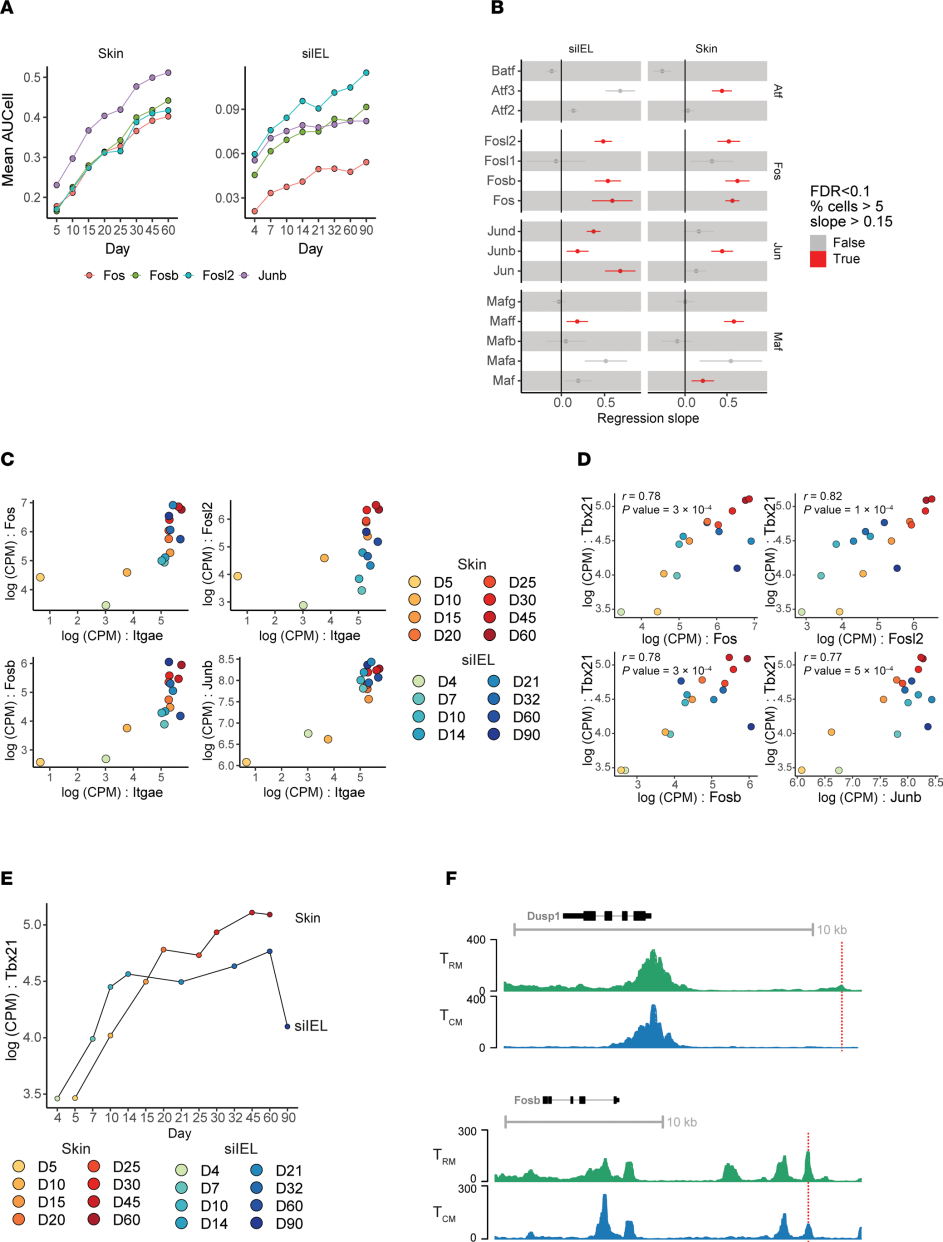
AP-1 transcription factor family members correlate with T_RM_ development. (**A**) Mean SCENIC Aucell scores for select regulons over time in the skin (left) and siIEL (right). Each line represents a unique regulon and the points represent the mean AUCell score for the regulon at the experimental timepoint. (**B**) linear modeling of AP-1 subfamilies in skin and siIEL. Each row is a gene with dots indicating the regression slope and 95% confidence interval from linear modeling of expression over time. Color indicates if it met our criteria to be considered a temporal-T_RM_ gene (FDR < 0.1, % cells > 5, regression slope > 0.15). (**C**) Expression of Fos-family genes and *Junb* versus *Itgae* over time in skin and siIEL. Each point represents a sample detailed in the legend that is shared with (**D**), and the *x*- and *y*-axes represent the Log(CPM) of *Itgae* and Fos family members, respectively. (**D**) Scatter plots showing the Log(CPM) of *Tbx21* on the *y*-axis and Log(CPM) of *Fosl2*, *Fos*, *Fosb*, and *Junb* on the *x* axis across skin and siIEL timepoints. Color scale indicates both anatomical location and experimental timepoint from which the sample came from. *r* and *P* values are from Pearson correlation. (**E**) *Tbx21* expression in skin and siIEL over time. The *x* axis represents time while the *y* axis represents Log(CPM) of *Tbx21*. Dots are connected by their neighboring timepoints. (**F**) ATAC-seq tracks from our T_RM_ and T_CM_ samples at the *Dusp1* and *Fosb* loci. Dotted line represents location of predicted Fos binding motif enriched in T_RM_ versus T_CM_.

Interestingly, the SCENIC analysis identified 12 regulons common to both skin and siIEL T_RM_ development, including the AP-1 transcription factor members *Fos* (skin *P* value = 1.5 × 10^–5^; siIEL *P* value = 7 × 10^–4^)*, Fosb* (skin *P* value = 3 × 10^–6^; siIEL *P* value = 2 × 10^–4^)*, Fosl2* (skin *P* value = 1.4 × 10^–5^; siIEL *P* value = 1 × 10^–4^) and *Junb* (skin *P* value = 3 × 10^–5^; siIEL *P* value = 0.008) (Figure 5A and Supplemental Table 6). In addition to being significant in both skin and siIEL, these AP-1 members had the highest regression slopes of any regulons in skin (Supplemental Figure 6B). When looking at the transcription factor subfamilies that encompass the proteins that are a part of functional AP-1 dimers, those of the Fos and Jun family were most consistently upregulated in T_RM_ (Figure 5B). The expression of AP-1 members also correlated with *Itgae* in the skin and siIEL (Figure 5C). A prevailing hypothesis about the role of AP-1 transcription factor members in T_RM_ development is that they coordinate the downregulation of the transcription factor T-bet (encoded by the gene *Tbx21*) (9, 45). Surprisingly, however, *Tbx21* itself met our stringent criteria to be included in our temporal T_RM_ signature (skin: regression slope = 0.32, *P* value = 4.39 × 10^–10^; siIEL: regression slope = 0.15, *P* value = 0.002). Furthermore, expression of *Tbx21* showed a strong positive correlation with the AP-1 family members *Fos* (*r* = 0.78, *P* value = 3 × 10^–4^), *Fosl2* (*r* = 0.82, *P* value 1 × 10^–4^), *Fosb* (*r* = 0.78, *P* value = 3 × 10^–4^) and *Junb* (*r* = 0.77, *P* value = 5 × 10^–4^) across both skin and siIEL, suggesting the proposed negative regulation of T-bet by AP-1 members to enable T_RM_ development is unlikely (Figure 5, D and E). Our ATAC-seq analysis identified open-chromatin sites in fully mature T_RM_ around genes from our temporal T_RM_ signature. While many open-chromatin sites were shared with T_CM_, there were specific peaks enriched in T_RM_ with predicted *Fos/Junb* binding motifs (Figure 5F, red lines).

We next looked for expression of AP-1 family members in human T_RM_ in the context of tissue transplantation. When comparing the host versus donor CD8^+^ T cells 796 days after allogenic-HSCT (37), we saw increased *JUNB* expression in the host cells, which should represent a bona fide population of T_RM_ (Supplemental Figure 6C). We did not see an increase in other AP-1 family members, suggesting that *JUNB* might be uniquely important in human skin T_RM_. In addition, Fitzpatrick et al. performed scRNA-seq on donor-derived T cells from an intestinal transplant recipient 1 year after transplantation (46). When reanalyzing this data, we observed that the CD8^+^ T cells from this dataset show robust expression of many AP-1 members (Supplemental Figure 6D).

**Figure 6:**
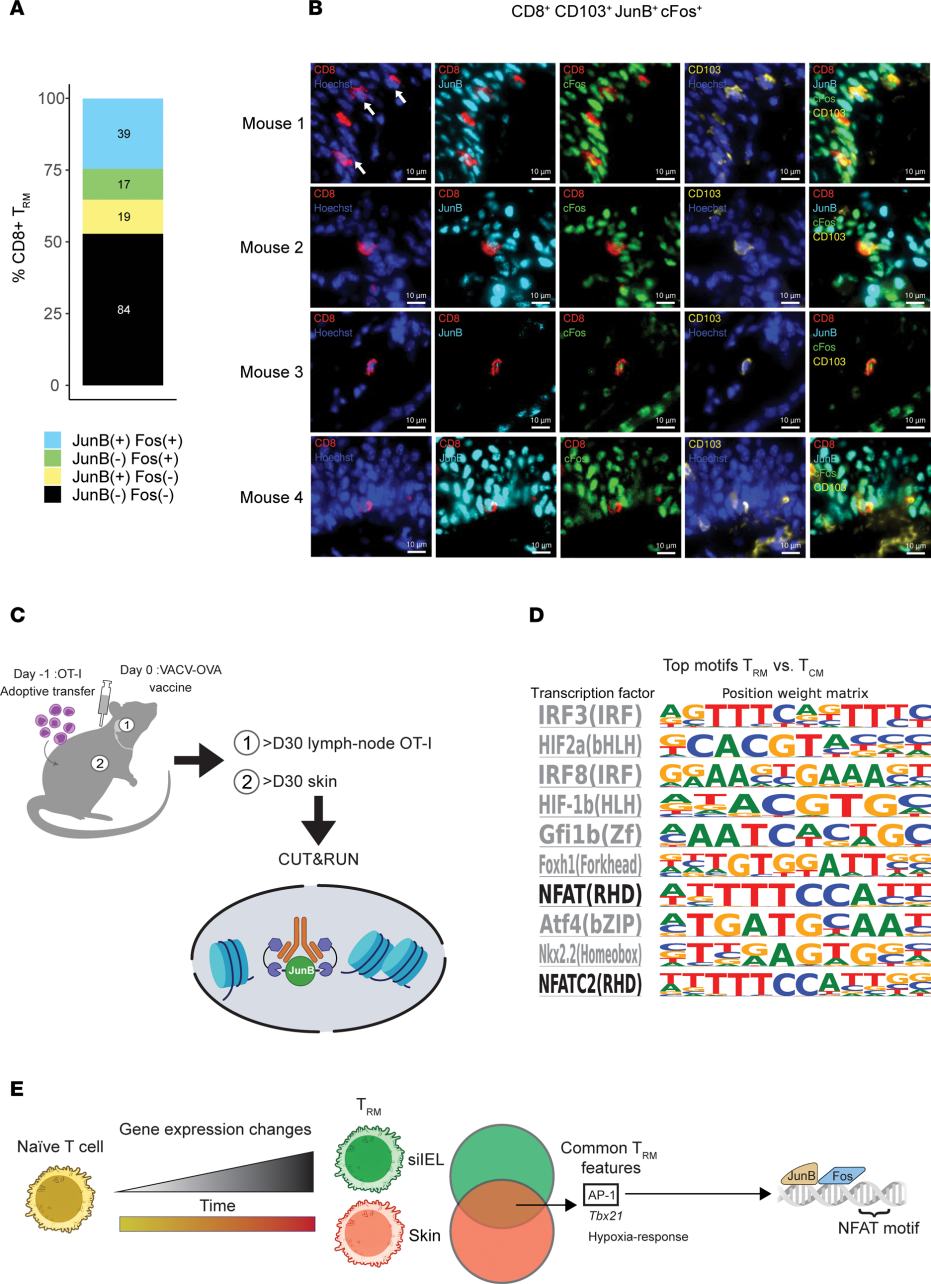
AP-1 is located in the nucleus of T_RM_ and are bound to sites of NFAT motifs. (**A**) Quantification of CD8^+^CD103^+^ skin T_RM_ as detected by t-CyCIF, broken down by the presence of cFos and JunB staining. The numbers in the boxes represent the number of cells in each category. 159 CD8^+^ CD11c^–^ cells were identified from the tail and ear skin from 5 mice. (**B**) t-CyCIF images of mouse tail skin epidermis 154 days after rVACV-OVA vaccination. Duplex and composite images of highlighted CD8^+^ T_RM_ cell expressing CD8 (red), JunB (cyan), and cFos (green) and CD103 (yellow)**.** Arrows indicate T_RM_ cells with positive JunB and cFos staining when multiple cells are in the same field of view. Scale bars: 10 μm.(**C**) Schematic of the CUT&RUN experimental design. Schematic created using BioRender (https://biorender.com). (**D**) HOMER-known motif analysis comparing T_RM_ and T_CM_ CUT&RUN samples profiled. Shown are the transcription factors and position weight matrices for the top 10 known motifs for T_RM_. NFAT motifs are bolded. (**E**) Summary of major findings.

We next endeavored to visualize AP-1 family members in T_RM_ by CyCIF (25). Specifically, we used high-plex tissue imaging to assess the expression of CD8, CD11c, CD103, cFos, and JunB to determine whether AP-1 family member proteins could be identified in skin OT-I T_RM_ cells more than 100 days after infection and whether they were localized to the cytoplasm or the nucleus (Figure 6, A and B). We were able to profile 159 CD8^+^CD103^+^ T cells, of which 36.5% (*n* = 58 cells) and 35.2% (*n* = 56 cells) stained for JunB and cFos, respectively. Of the cells that expressed either AP-1 member, 52% (*n* = 39 cells) expressed both JunB and cFos. While CD8 and CD103 were clearly diffusely expressed, consistent with membrane localization, JunB and cFos staining was generally found in the nucleus (colocalized with the nuclear Hoechst stain). To date, this is the first demonstration of expression of JunB and cFos in the nucleus of resting memory T cells. This suggests that AP-1 complexes are preformed and poised to be activated in T_RM_.

The nuclear localization of AP-1 members suggested these transcription factors are DNA-bound. Therefore, we pursued CUT&RUN (cleavage under targets and release using nuclease) to identify AP-1–DNA binding sites in T_RM_ while profiling T_CM_ as a control (Figure 6C). Specifically, we targeted JunB, given that our analyses suggest it’s importance in both human and mouse skin T_RM_. We found motifs for multiple Interferon regulatory factors (IRF) transcription factors enriched in T_RM_ (Figure 6D). Additionally, there was an enrichment of JunB binding at nuclear factor of activated T cells (NFAT) motifs in T_RM_, consistent with the hypothesis that these cells are poised to be activated in a recall response.

## Discussion

In this study, we sought to carefully examine the ontogeny and development of different memory T cells arising from a common naive T cell population by performing a scRNA-seq time course analysis of CD8^+^ OT-I T cells after a skin infection with VACV. To strengthen our study, we also incorporated an analogous scRNA-seq time course dataset that looked at LCMV-specific T cells in the siIEL. Our major findings are summarized in Figure 6E.

Our analysis of cells isolated from the dLN showed that T cell subsets derived from naive cells showed cellular diversification as early as day 5. Intriguingly, one of the three dLN subsets (C10), found predominantly at day 5, exclusively expressed *Fut7*, which has been shown to be necessary for T cell trafficking to the skin, leading us to speculate that this population represents the cells destined to for tissue homing (47). Additionally, the gene expression profile of C10 most closely correlated with skin clusters C2, C3, and C6, supporting the hypothesis that these are skin-trafficking cells. In contrast, among the early dLN populations, C8 showed the highest expression of *Sell* as well as *Btg1*, a gene recently associated with T cell quiescence (31). These results support the hypothesis that activation in the dLN leads to the generation of different T cell subsets, some of which appear to be destined to traffic to the skin, while others are destined to remain in the lymph node or recirculate between blood and dLN.

Our comparative analysis of transcriptional programs driving T_RM_ ontogeny across anatomical sites found 506 and 248 genes that were unique to siIEL and skin compartments, respectively. The siIEL-specific genes were enriched for heat-shock proteins (*Hspa1a*, *Hspa1b*, *Hsph1*, *Hsp90aa1*, *Dnaja4*). While never described in this context, heat-shock proteins are known to play a role in response to hypoxia (48), an external cue that has been linked to T_RM_ development (49). Whether the upregulation of these genes in the LCMV model is related to hypoxic signaling or other external factors remains to be understood. In parallel, *Ikzf2* (encodes Helios) was identified as a skin-specific T_RM_ development transcription factor, which is best known for its ability to modulate regulatory T cell effector function and identity (50, 51). Nonetheless, though *Ikzf2* was uniquely expressed in the skin dataset, we cannot rule out that this distinction was due to differences in the infectious agents used to induce T_RM_ phenotypes (VACV versus LCMV). Indeed, an *IKZF2*-expressing population of T_RM_ cells has been described in the human intestine (46). The T_RM_ cells isolated from the skin following VACV infection are those from the site of primary infection, where differentiation is driven in part by acute tissue inflammation. In contrast, the T_RM_ cells in the small intestine that arise from LCMV infection delivered intraperitoneally represent those from a secondarily populated site, likely with much less inflammation. Such distinctions in the 2 infectious models that were analyzed could contribute to the differences in T_RM_-associated genes across tissue compartments.

Despite the differences in experimental design, tissues analyzed, and viruses used, 136 genes were commonly associated with T_RM_ development in both skin and siIEL. Of those 136 genes, 100 were defined as our temporal T_RM_ signature, after removing the 36 memory genes also associated with T_CIRC_ populations. Previously reported T_RM_ signatures were derived from the comparison of T_RM_ to circulating memory T cells (6, 16). While these comparisons are valuable, our analysis indicated that the dominant gene expression changes leading to T cell memory happen gradually over time, rather than in a phasic manner. Using a linear model approach enabled us to capture this dominant pattern and define T_RM_ differentiation at higher granularity by considering the effector T cell phase as our baseline. Additionally, our approach enabled us to compare the results of T_RM_ to that of T_CIRC_ to find genes common among different mature memory T cell fates, regardless of circulatory capacity. Encouragingly, we did find overlap between our 100-gene temporal T_RM_ signature and those previously reported (6, 16). These included genes such as *Itgae*, *Nr4a3*, and *Rgs1*, all of which have been shown to play a role in T_RM_ formation (6, 52). It should be noted that our temporal signature lacks certain factors previously defined as T_RM_-specific genes, such as *Runx3*, *Notch1*, *Znf683* (encodes Hobit)*,* and *Prdm1* (encodes Blimp1). *Runx3* was found to be a T_RM_-associated gene in the siIEL compartment, but not in skin. *Notch1* was found to have low expression in both the skin (8.7% of cells across all timepoints) and siIEL (11.7% of cells across all timepoints) datasets, and its association with T_RM_ development has been best described in the lung (40), suggesting it may be a tissue-specific driver of T_RM_ differentiation. *Prdm1* and *Zfp683* were both expressed at very low levels in our skin cells (*Prdm1:* 4% across all timepoints, *Zfp683*: 0.8% across all timepoints), making it difficult to evaluate their contribution in driving T_RM_ development through our linear modeling approach. However, it should be noted that *Prdm1* did meet our significance threshold for our siIEL T_RM_ gene list (regression slope = 0.199, *P* value = 3.2 × 10 ^–7^). Importantly, our 100-gene T_RM_ signature also includes genes uniquely captured by our linear modeling approach (e.g., *Fosb*, *Id3*, *Cebpb*) that could successfully distinguish resident from circulating T cell subsets, further validating our analytical strategy. Given our model’s ability to find new genes previously undescribed by other gene sets while also lacking genes known to be important for T_RM_ development, we view the temporal signature as additive to the current field of knowledge. Future work will be needed to characterize the role of these newly defined targets in T_RM_ development.

As noted above, this temporal T_RM_ signature was hallmarked by AP-1 transcription factor members, whose importance in T_RM_ differentiation across tissues was also highlighted in our SCENIC analysis. Additionally, motifs corresponding to AP-1 binding sites were found to be enriched in the open chromatin of T_RM_ when compared to T_CM_. The importance of AP-1 family members in T_RM_ development have been described recently in the siIEL (9) and skin (53), and our analyses confirm that this is likely a generalizable marker of T_RM_ development and maintenance. However, our study is the first to show AP-1–member protein nuclear staining in resting T_RM_ cells. In addition, this is also the first study to show that human T_RM_ distinctively express AP-1 transcription factor members. While multiple AP-1 members were expressed in human siIEL, *JUNB* was most specific for T_RM_ in skin. One concern regarding the upregulation of AP-1 family members is their ability to be induced by heat-based tissue digestions (54). While we cannot dispute that heat digestion is a mechanism for AP-1 upregulation, our linear modeling that produced our T_RM_ signature inherently controls for this, as all samples across time were digested equivalently. In addition, the upregulation of *JUNB* in the host CD8^+^ T cells when compared to donor cells from an allogenic-HSCT patient cannot solely be attributed to digestion, as they were all from the same biopsy. One limitation of finding AP-1 members in the T_RM_ in the context of tissue transplantation is that we cannot rule out other factors inherent to that setting, such as altered activation and immunosuppression. However, our CyCIF staining of AP-1 members in resting T_RM_ also highlights the expression of these transcription factors outside the context of other technical factors. One proposed hypothesis for the role of AP-1 transcription factors in T_RM_ differentiation is that it can suppress T-bet expression, a reported necessary step for T_RM_ development (15). However, in both the skin and siIEL tissue niches, we saw a strong correlation between AP-1 family members and T-bet expression levels (encoded by the *Tbx21* gene). Given this observation, it remains unclear what role AP-1 plays in T_RM_ differentiation and maintenance, and future mechanistic studies that alter expression of AP-1 members in T_RM_ are necessary to provide clarity.

This is a potentially novel observation yielding a new perspective on AP-1 members in mature T_RM_ biology. In nonadaptive immunity settings in murine epidermal stem cells, constitutively expressed AP-1 members (some bound to DNA at rest) have been shown to be critical to orchestrate inflammatory memory, leaving keratinocytes poised for rapid recall responses (55). AP-1 has been shown to be important during initial T cell activation, directing chromatin remodeling of naive T cells (56). There is also good evidence that T cell receptor ligation by antigen in the context of MHC results in calcium influx and nuclear translocation of NFAT family members (57). NFAT–AP-1 complexes involving contiguous TF factor binding sites are some of the most potent known superenhancers of T cell cytokines and effector functions (57–61). Given this, it is tempting to hypothesize that constitutive AP-1 expression and its nuclear localization enable T_RM_ cells to be “poised” for the rapid recall immune responses after TCR engagement alone, which results in NFAT nuclear translocation and formation of the NFAT/AP-1 transcription enhancer complex. Thus, rather than simply being involved in the development of T_RM_, AP-1 members are critical to the unique functional characteristic of T_RM_, including rapid recall. If true, this is a previously unreported mode of memory T cell activation, which borrows from innate immune memory.

## Methods

### Sex as a biological variable.

The majority of mice used in this study were male C57BL/6J mice. Sex was not considered as a biological variable.

### Mice.

WT C57BL/6J mice were purchased from Jackson Laboratory. OT-I/Rag1^–/–^ /Thy1.1 mice were bred and maintained in the animal facility of Harvard Institute of Medicine, Harvard Medical School.

### Adoptive transfer and viral infection.

For adoptive transfer, lymph nodes were collected from naive OT-I/Rag1^–/–^ /Thy1.1 mice at the age of 6–8 weeks. OT-I T cells were then purified by negative magnetic cell sorting using mouse CD8α^+^ T-cell isolation kit (130-104-075; Miltenyi Biotec) according to the manufacture’s protocols. Purified OT-I cells were then transferred intravenously into gender-matched C57BL/6J recipient mice at the number of 5 × 10^5^ cells per mice.

VACV-OVA was a gift from Bernard Moss (NIH, Bethesda, Maryland, USA). VACV-OVA stocks were expanded in Hela cells (American Tissue Culture Company) and titrated in CV-1 cells (American Tissue Culture Company) by standard procedures. VACV-OVA were infected to mice at 2 × 10^6^ PFU/mice by skin scarification (s.s.) on ear and tail as described previously (12, 13).

### Flow cytometry, cell sorting, and imaging flow cytometry.

Single cell suspensions were prepared as described before. Briefly, for lymph nodes, tissue specimens were mashed through 70 μm cell strainers before being lysed with RBC lysis buffer (00-4333-57; eBioscience). For skin, tail skin as well as separated dorsal and ventral halves of ear skin were minced and digested at 37°C for 30 minutes, with HBSS solution with 1 mg/mL collagenase A (11088785103; Roche) and 40 μg/mL DNase I (10104159001; Roche) before being filtered through 70 μm cell strainers. Cells were washed 3 times and kept in PBS supplemented with 2% FBS.

For flow cytometry, digested and purified skin single cells suspensions were stained and loaded onto FACSCanto II (BD Biosciences) for analysis or FACSAria (BD Biosciences) for sorting. To isolate OT-I cells for scRNA-seq, cells were stained with antibodies against mouse CD8a (100714; Biolegend) and CD90.1 (202518, Biolegend). For the isolation of T_CM_ cells, cells were also stained with an antibody against CD62L (560514; BD). FACS data were analyzed with Flowjo software (Tree Star).

### T-CyCIF imaging.

FFPE sections of mouse tail and ear skin were prepared and t-CyCIF was performed as previously reported (25, 62) following the published protocol on protocols.io (dx.doi.org/10.17504/protocols.io.bjiukkew). Slides were stained with Hoechst 33342 (0.25 μg/mL; LI-COR Biosciences) and antibodies against CD8a (83012BC; Cell Signaling Technologies), CD103 (AF1990; R&D Systems), CD11c (64675BC; Cell Signaling Technologies), JunB (3753; Cell Signaling Technologies), and cFos (sc-166940 AF647; Santa Cruz Biotechnologies) in SuperBlockTM Blocking Buffer. Images were acquired using the CyteFinder II HT Instrument (RareCyte Inc. Seattle WA) with a 20x/0.75 NA objective. ASHLAR (Alignment by Simultaneous Harmonization of Layer/Adjacency Registration) software was used to stitch the image tiles and register each immunofluorescence cycle together into a single OME-TIFF file. For quantification, 5 tails and 8 ears from 5 mice were manually analyzed for CD8^+^ CD11c^–^ cells expressing CD103, JunB, and cFos.

### scRNA-seq.

For the scRNA-seq profiling, live CD8a^+^CD90.1^+^ cells were sorted as described above and then approximately 12,000 single cells were loaded to each 10X channel with a recovery goal of 6,000 single cells. Cell suspensions were loaded along with reverse transcriptase reagents, 3′ gel beads, and emulsification oil onto separate channels of a 10X Single Cell B Chip, which was loaded into the 10X Chromium instrument to generate emulsions. Emulsions were transferred to PCR strip tubes for immediate processing and reverse transcription. Library preparation was performed according to manufacturer’s recommendations. Expression libraries were generated using the Chromium Single Cell 3′V3 chemistry (10X Genomics PN-120262). DNA and library quality was evaluated using an Agilent 2100 Bioanalyzer and concentration was quantified using the Qubit dsDNA high-sensitivity reagents (Thermo Fisher Scientific). Gene expression libraries were sequenced on an Illumina NextSeq instrument using the Illumina NextSeq 500/550 with the following sequencing configuration: Read 1=28, Read 2=56, index 1=8, index 2=0.

### CUT&RUN.

Two groups of 20 mice received 1 × 10^6^ Thy1.1 congenic OT-I cells by retroorbital injection 1 day before VACV-OVA infection on the tail and ears. On day 30 and day 45 after infection, the tails, ears, and draining lymph nodes were prepared for cell sorting. Cells were sorted based on live/dead discrimination dye, CD45^+^TCRb^+^CD8^+^CD4^–^Thy1.1^+^. From the skin, 27,600 and 16,600 OT-I cells were isolated on day 30 and day 45, respectively. 350,000 and 500,000 OT-I cells were sorted from the lymph nodes on day 30 and 45, respectively. After sorting, cells were immediately put on ice. Cells were then resuspended in pre-chilled FBS+10% DMSO and transferred to a Mr. Frosty Cryo Freezing Container and placed in a –80°C freezer. Cells were sent to Active Motif for CUT&RUN analysis against JunB (Cell signaling; clone C37F9). To increase the signal from JunB, OT-I cells from the two time points were pooled.

### Statistics.

All statistics for this study were calculated in R. All *P* values were corrected with a false discovery rate using using the Benjamini-Hochberg method and findings with an FDR < 0.1 were considered significant. All tests of significance were 2-sided. Details on how *P*-values were calculated for all computational analyses can be found in our Supplemental methods. All differential gene expression *P* values were calculated with a Wald test on pseudobulk counts using the DESeq2 package in R. *P* values for SCENIC analyses were calculated with linear regression using the “lm” function in R. Empirical p-values via permutation were calculated for GSEA using the fgsea package in R.

### Study approval.

All animal experiments protocols were approved by the Institutional Animal Care and Use Committee at Brigham and Women’s Hospital. All animal experiments were conducted in accordance with the guidelines from the Center for Animal Resources and Comparative Medicine at Harvard Medical School.

### Data availability.

scRNA-seq count matrices and related data is deposited in the GEO database (under accession # GSE237735). Values for all data points in graphs are reported in the Supporting Data Values file.

### Code availability.

Source code for data analysis is available on GitHub (https://github.com/villani-lab/trm_development [branch: main, commit ID: e537bcb]). A full list of software packages and versions included in the analyses is included in Supplemental Table 7.

## Author contributions

TSK designed the overall study. YP, YY, JZ, TT, TP, CS, KW, and ALK performed the mice experiments and sample collection. KM generated the scRNA-seq data. YP and JZ helped generate the ATAC-seq data. Data analysis and interpretation was performed by NPS with substantial contributions from YY, YP, ACV, and TSK. ACV managed and supervised scRNA-seq data generation and analysis. YY performed follow-up validation experiments and immunophenotyping. JBW and SMP performed the CyCIF experiments with guidance from PKS. NPS, YY, ACV, and TSK wrote the manuscript with substantial revisions by JBW. All authors read or provided comments on the manuscript.

## Supplementary Material

Supplemental data

Supplemental tables 1-7

Supporting data values

## Version 1 06/23/2025

Electronic publication

## References

[1] Mosmann TR (1986). Two types of murine helper T cell clone. I. Definition according to profiles of lymphokine activities and secreted proteins. J Immunol.

[2] Sallusto F (1999). Two subsets of memory T lymphocytes with distinct homing potentials and effector functions. Nature.

[3] Rotrosen E, Kupper TS (2023). Assessing the generation of tissue resident memory T cells by vaccines. Nat Rev Immunol.

[4] Mackay LK (2016). Hobit and Blimp1 instruct a universal transcriptional program of tissue residency in lymphocytes. Science.

[5] Fonseca R (2022). Runx3 drives a CD8^+^ T cell tissue residency program that is absent in CD4^+^ T cells. Nat Immunol.

[6] Milner JJ (2017). Runx3 programs CD8^+^ T cell residency in non-lymphoid tissues and tumours. Nature.

[7] Parga-Vidal L (2021). Hobit identifies tissue-resident memory T cell precursors that are regulated by Eomes. Sci Immunol.

[8] Skon CN (2013). Transcriptional downregulation of S1pr1 is required for the establishment of resident memory CD8^+^ T cells. Nat Immunol.

[9] Kurd NS (2020). Early precursors and molecular determinants of tissue-resident memory CD8^+^ T lymphocytes revealed by single-cell RNA sequencing. Sci Immunol.

[10] Ariotti S (2012). Tissue-resident memory CD8^+^ T cells continuously patrol skin epithelia to quickly recognize local antigen. Proc Natl Acad Sci U S A.

[11] Liu L (2010). Epidermal injury and infection during poxvirus immunization is crucial for the generation of highly protective T cell-mediated immunity. Nat Med.

[12] Pan Y (2021). Epicutaneous immunization with modified vaccinia Ankara viral vectors generates superior T cell immunity against a respiratory viral challenge. NPJ Vaccines.

[13] Pan Y (2017). Survival of tissue-resident memory T cells requires exogenous lipid uptake and metabolism. Nature.

[14] Stolley JM (2020). Retrograde migration supplies resident memory T cells to lung-draining LN after influenza infection. J Exp Med.

[15] Mackay LK (2015). T-box transcription factors combine with the cytokines TGF-β and IL-15 to control tissue-resident memory T cell fate. Immunity.

[16] Mackay LK (2013). The developmental pathway for CD103(+)CD8^+^ tissue-resident memory T cells of skin. Nat Immunol.

[17] Jiang X (2012). Skin infection generates non-migratory memory CD8^+^ T(RM) cells providing global skin immunity. Nature.

[18] Milner JJ (2020). Heterogenous populations of tissue-resident CD8^+^ T cells are generated in response to infection and malignancy. Immunity.

[19] Parga-Vidal L (2022). Hobit and Blimp-1 regulate T_RM_ abundance after LCMV infection by suppressing tissue exit pathways of T_RM_ precursors. Eur J Immunol.

[20] Behr FM (2021). Circulating memory CD8^+^ T cells are limited in forming CD103^+^ tissue-resident memory T cells at mucosal sites after reinfection. Eur J Immunol.

[21] Woyciechowski S (2017). α_4_ β_1_ integrin promotes accumulation of tissue-resident memory CD8^+^ T cells in salivary glands. Eur J Immunol.

[22] Casey KA (2012). Antigen-independent differentiation and maintenance of effector-like resident memory T cells in tissues. J Immunol.

[23] Liao W (2020). Isolation of mouse kidney-resident CD8^+^ T cells for flow cytometry analysis. J Vis Exp.

[24] Beura LK (2015). Lymphocytic choriomeningitis virus persistence promotes effector-like memory differentiation and enhances mucosal T cell distribution. J Leukoc Biol.

[25] Lin J-R (2018). Highly multiplexed immunofluorescence imaging of human tissues and tumors using t-CyCIF and conventional optical microscopes. Elife.

[26] Jacomy M (2014). ForceAtlas2, a continuous graph layout algorithm for handy network visualization designed for the Gephi software. PLoS One.

[27] Ko ME (2020). FLOW-MAP: a graph-based, force-directed layout algorithm for trajectory mapping in single-cell time course datasets. Nat Protoc.

[28] Schiebinger G (2019). Optimal-transport analysis of single-cell gene expression identifies developmental trajectories in reprogramming. Cell.

[29] De Silva D (2021). Robust T cell activation requires an eIF3-driven burst in T cell receptor translation. Elife.

[30] Chen G (2018). Ezh2 regulates activation-induced CD8^+^ T cell cycle progression via repressing *Cdkn2a* and *Cdkn1c* expression. Front Immunol.

[31] Hwang SS (2020). mRNA destabilization by BTG1 and BTG2 maintains T cell quiescence. Science.

[32] Lange M (2022). CellRank for directed single-cell fate mapping. Nat Methods.

[33] Rao RR (2010). The mTOR kinase determines effector versus memory CD8^+^ T cell fate by regulating the expression of transcription factors T-bet and Eomesodermin. Immunity.

[34] Ishifune C (2019). Regulation of membrane phospholipid asymmetry by Notch-mediated flippase expression controls the number of intraepithelial TCRαβ^+^CD8αα^+^ T cells. PLoS Biol.

[35] Kaech SM (2003). Selective expression of the interleukin 7 receptor identifies effector CD8 T cells that give rise to long-lived memory cells. Nat Immunol.

[36] Yang CY (2011). The transcriptional regulators Id2 and Id3 control the formation of distinct memory CD8^+^ T cell subsets. Nat Immunol.

[37] de Almeida GP (2022). Human skin-resident host T cells can persist long term after allogeneic stem cell transplantation and maintain recirculation potential. Sci Immunol.

[38] Chennareddy S (2025). Single-cell RNA sequencing comparison of CD4^+^, CD8^+^ and T-cell receptor γδ^+^ cutaneous T-cell lymphomas reveals subset-specific molecular phenotypes. Br J Dermatol.

[39] Boland BS (2020). Heterogeneity and clonal relationships of adaptive immune cells in ulcerative colitis revealed by single-cell analyses. Sci Immunol.

[40] Hombrink P (2016). Programs for the persistence, vigilance and control of human CD8^+^ lung-resident memory T cells. Nat Immunol.

[41] Aibar S (2017). SCENIC: single-cell regulatory network inference and clustering. Nat Methods.

[42] Van de Sande B (2020). A scalable SCENIC workflow for single-cell gene regulatory network analysis. Nat Protoc.

[43] Chen Y (2021). Single-cell transcriptomics reveals core regulatory programs that determine the heterogeneity of circulating and tissue-resident memory CD8^+^ T cells. Cells.

[44] Sullivan JA (2012). FOXO3 regulates the CD8 T cell response to a chronic viral infection. J Virol.

[45] Yenyuwadee S (2022). The evolving role of tissue-resident memory T cells in infections and cancer. Sci Adv.

[46] FitzPatrick MEB (2021). Human intestinal tissue-resident memory T cells comprise transcriptionally and functionally distinct subsets. Cell Rep.

[47] Aires DJ (2019). T-cell trafficking plays an essential role in tumor immunity. Lab Invest.

[48] Baird NA (2006). Induction of the heat shock pathway during hypoxia requires regulation of heat shock factor by hypoxia-inducible factor-1. J Biol Chem.

[49] Hasan F (2021). Hypoxia acts as an environmental cue for the human tissue-resident memory T cell differentiation program. JCI Insight.

[50] Getnet D (2010). A role for the transcription factor Helios in human CD4(+)CD25(+) regulatory T cells. Mol Immunol.

[51] Thornton AM (2019). Helios^+^ and Helios^–^ Treg subpopulations are phenotypically and functionally distinct and express dissimilar TCR repertoires. Eur J Immunol.

[52] Gebhardt T (2009). Memory T cells in nonlymphoid tissue that provide enhanced local immunity during infection with herpes simplex virus. Nat Immunol.

[53] Buquicchio FA (2024). Distinct epigenomic landscapes underlie tissue-specific memory T cell differentiation. Immunity.

[54] Crowl JT (2022). Tissue-resident memory CD8^+^ T cells possess unique transcriptional, epigenetic and functional adaptations to different tissue environments. Nat Immunol.

[55] Larsen SB (2021). Establishment, maintenance, and recall of inflammatory memory. Cell Stem Cell.

[56] Yukawa M (2020). AP-1 activity induced by co-stimulation is required for chromatin opening during T cell activation. J Exp Med.

[57] Macián F (2002). Transcriptional mechanisms underlying lymphocyte tolerance. Cell.

[58] Mognol GP (2019). Targeting the NFAT:AP-1 transcriptional complex on DNA with a small-molecule inhibitor. Proc Natl Acad Sci U S A.

[59] Wisniewska MB (2007). Dimer composition and promoter context contribute to functional cooperation between AP-1 and NFAT. J Mol Biol.

[60] Johnson BV (2004). Granulocyte-macrophage colony-stimulating factor enhancer activation requires cooperation between NFAT and AP-1 elements and is associated with extensive nucleosome reorganization. Mol Cell Biol.

[61] Boise LH (1993). The NFAT-1 DNA binding complex in activated T cells contains Fra-1 and JunB. Mol Cell Biol.

[62] Burger ML (2021). Antigen dominance hierarchies shape TCF1^+^ progenitor CD8 T cell phenotypes in tumors. Cell.

