# Supplementary material for: Resident memory T cell development is gradual and shows AP-1 gene expression in mature cells
Source: JCI Insight. 2025 Jun 23;10(12):e187381. doi: 10.1172/jci.insight.187381 (PMC12220954; doi:10.1172/jci.insight.187381)
Supplement: Supplemental data [file jciinsight-10-187381-s080.pdf]

Supplemental Figure 1

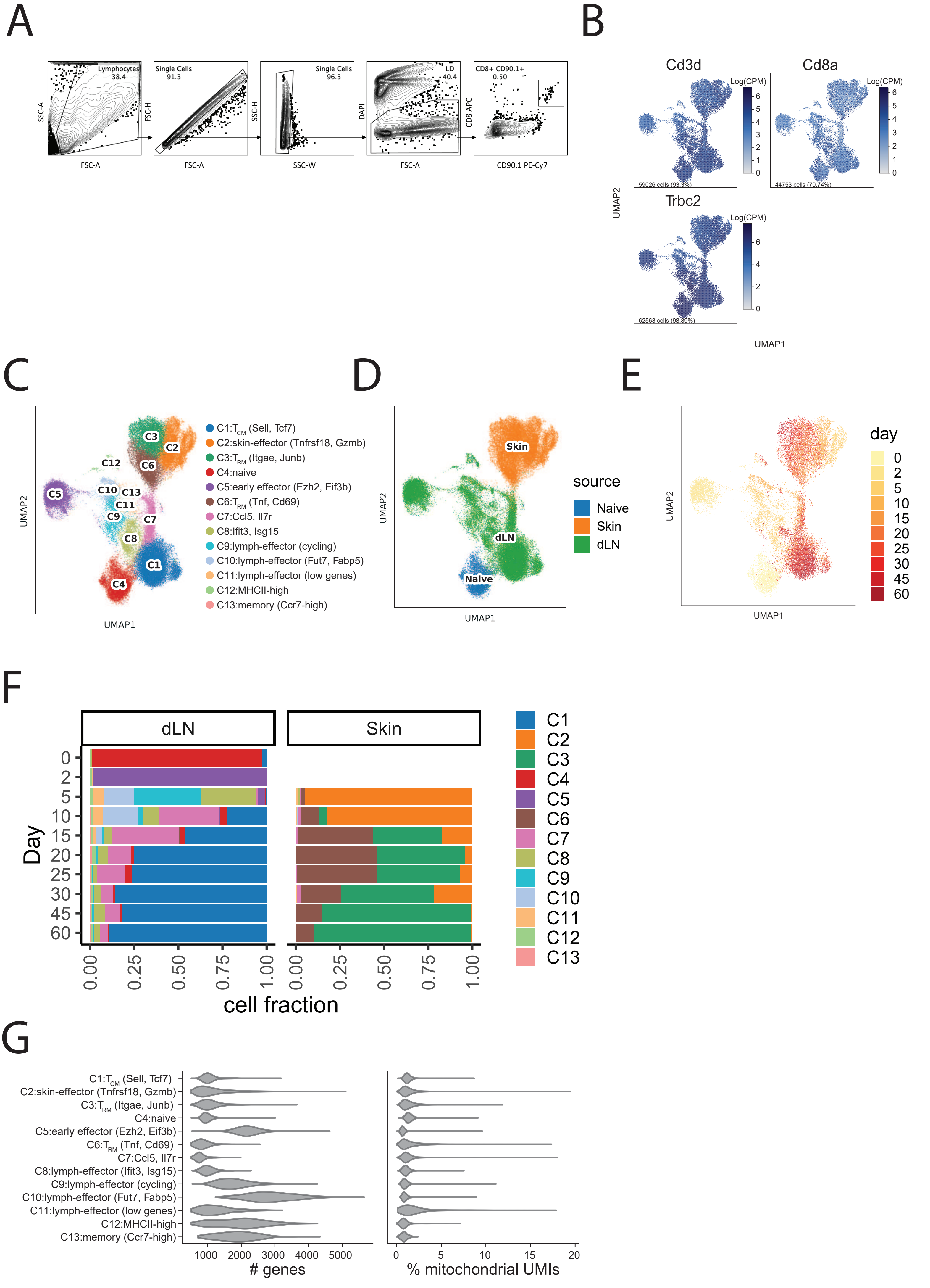

Supplemental Figure 1. scRNAseq processing and QC metrics. (A) Gating strategy used to isolate live $CD8^{+}CD90.1^{+}$ cells for downstream scRNAseq profiling. **(B)** UMAP embedding feature plots of 63,265 high-quality single cells, using color to represent gene expression levels (Log(CPM)) of canonical T cell genes (*Cd3d, Cd8a, Trbc2*). Cell number and percentage represent expression across all cells. **(C)** UMAP embedding showing predicted Leiden clusters listed on the right. **(D)** UMAP embedding of cells pseudocolored by tissue source. **(E)** UMAP embedding of cells pseudocolored by experimental timepoint. **(F)** Cluster composition at every timepoint. Bars represent the fraction of cells in dLN (left) and skin (right) that were assigned to the corresponding clusters. **(G)** Distribution of the number of captured genes (left) and percentage of mitochondrial unique molecular identifiers (UMIs) (right) in the cells across all clusters.

Supplemental Figure 2

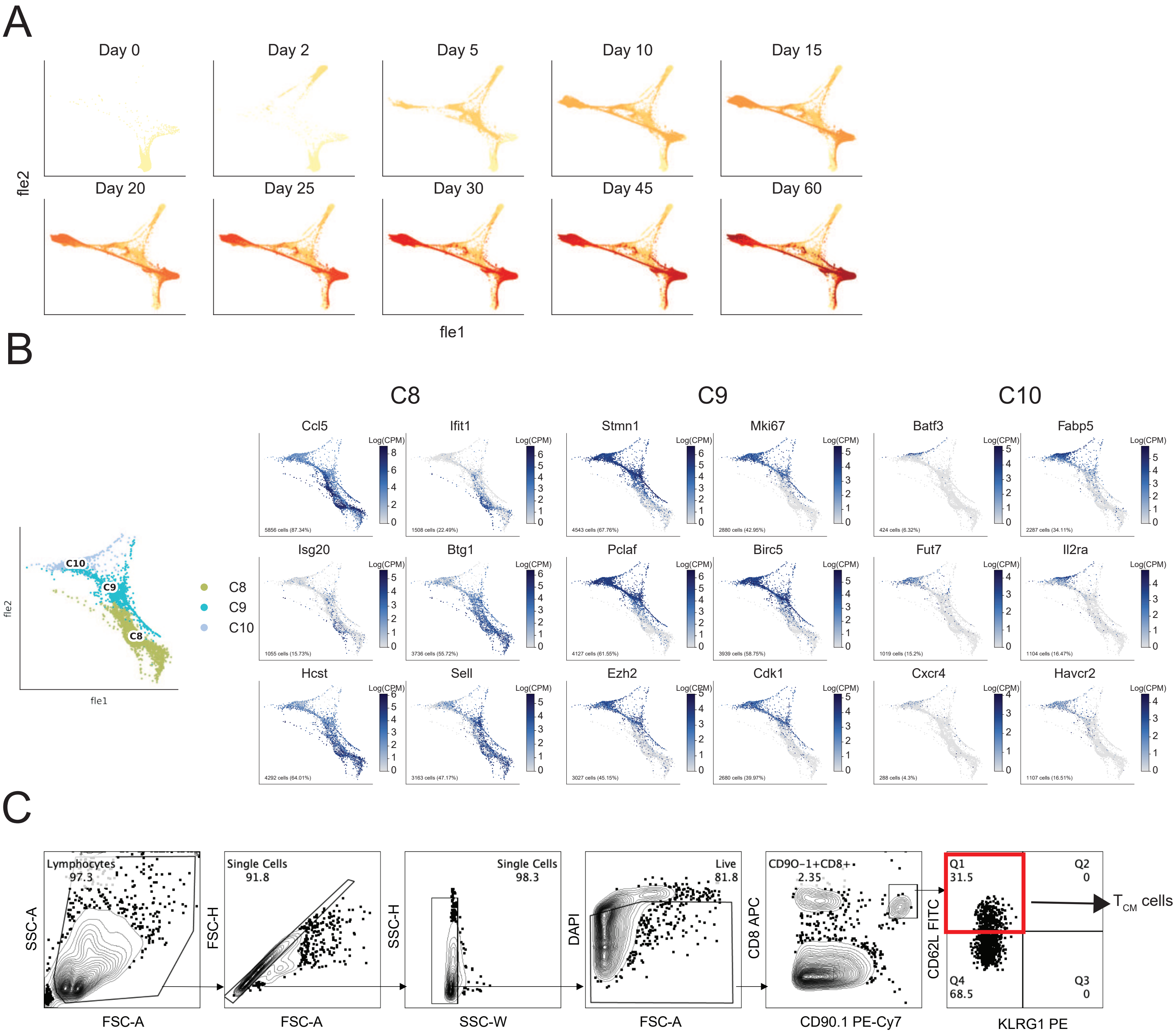

Supplemental Figure 2. Early heterogeneity of T cell post-infection (A) Force-directed layout embedding (FLE) of single-cells colored by timepoint. Each plot in the panel represents the cells from that timepoint and all previous timepoints. **(B)** (left) FLE of clusters most associated with day 5 dLN cells (C8, C9, and C10). (right) Feature plots using color to represent gene expression levels (Log(CPM)) of genes associated with each cluster. Cell number and percentage represent expression across all cells in C8, C9 and C10. **(C)** Gating strategy for the isolation of T_{CM} cells used for ATACseq profiling experiment. Gated T_{CM} cells are highlighted in the red box.

Supplemental Figure 3

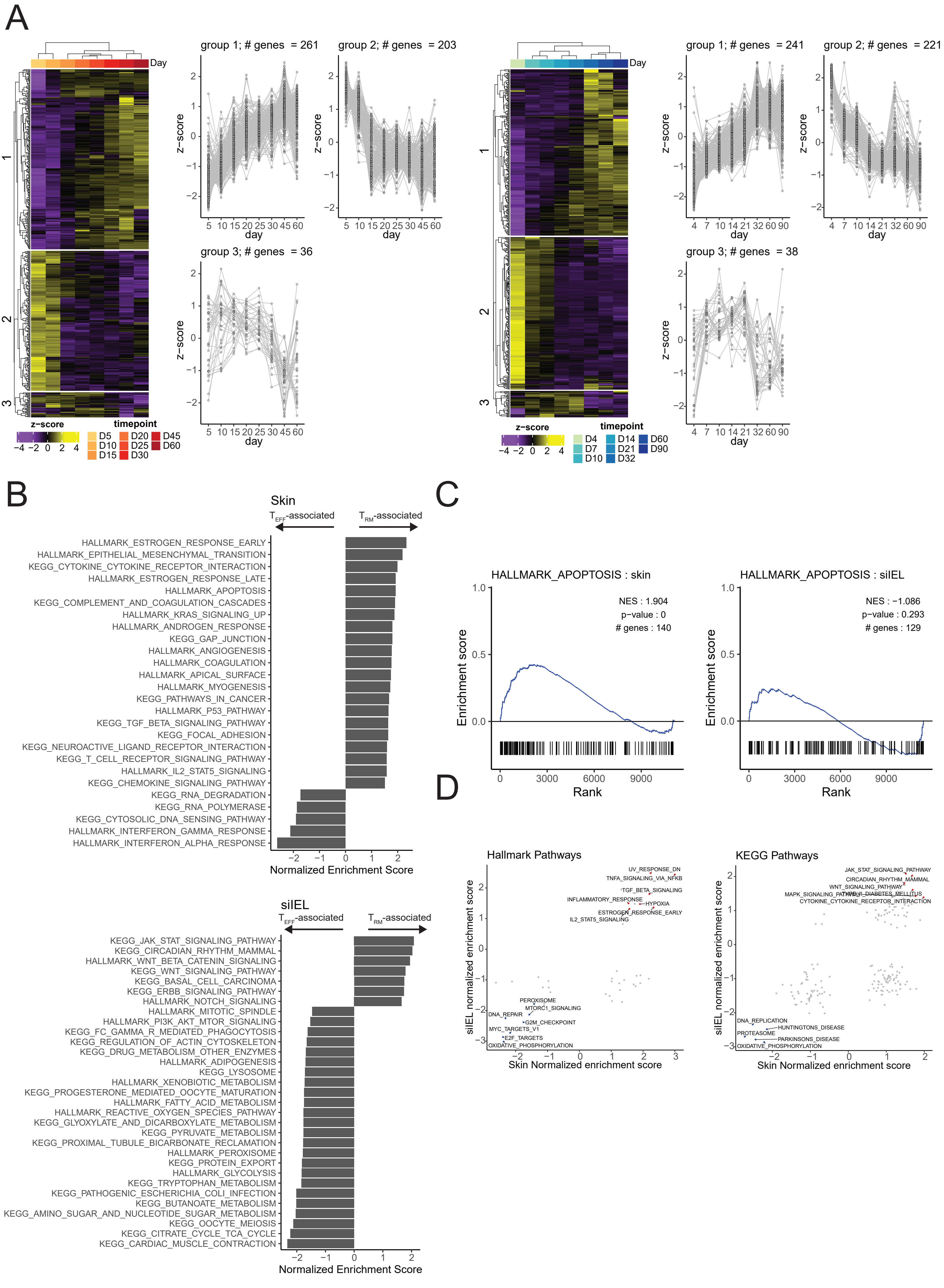

Supplemental Figure 3. The dominant pattern of gene expression change in T cells post-infection is gradual over time. (A) Top 500 genes in the skin (left) and siIEL (right) datasets by variance, plotted over time. Genes in the heatmap are broken up into three groups as determined by hierarchical clustering and are displayed as line plots to the right. **(B)** Gene set enrichment analysis (GSEA) of both skin and siIEL temporal analysis was performed using the KEGG and HALLMARK gene set libraries. Plots show the normalized enrichment scores (NES, x-axis) of all gene sets that were uniquely significant in skin (top) and siIEL (bottom) (FDR < 0.1). Positive NES represents gene sets associated with later timepoints (i.e. T_{RM}) and negative NES represents gene sets associated with earlier timepoints (i.e. Effector T cells). **(C)** GSEA of the HALLMARK apoptosis gene signature for skin (top) and siIEL (bottom) T_{RM} genes. **(D)** Gene set enrichment analysis results for skin and siIEL using the HALLMARK (left) and KEGG (right) pathways. Each point represents a gene set and the x- and y-axes represent the skin and siIEL normalized enrichment scores respectively. Labelled points highlighted in red and blue show gene sets that were significantly up-regulated and down-regulated respectively in both skin and siIEL (FDR < 0.1).

Supplemental Figure 4

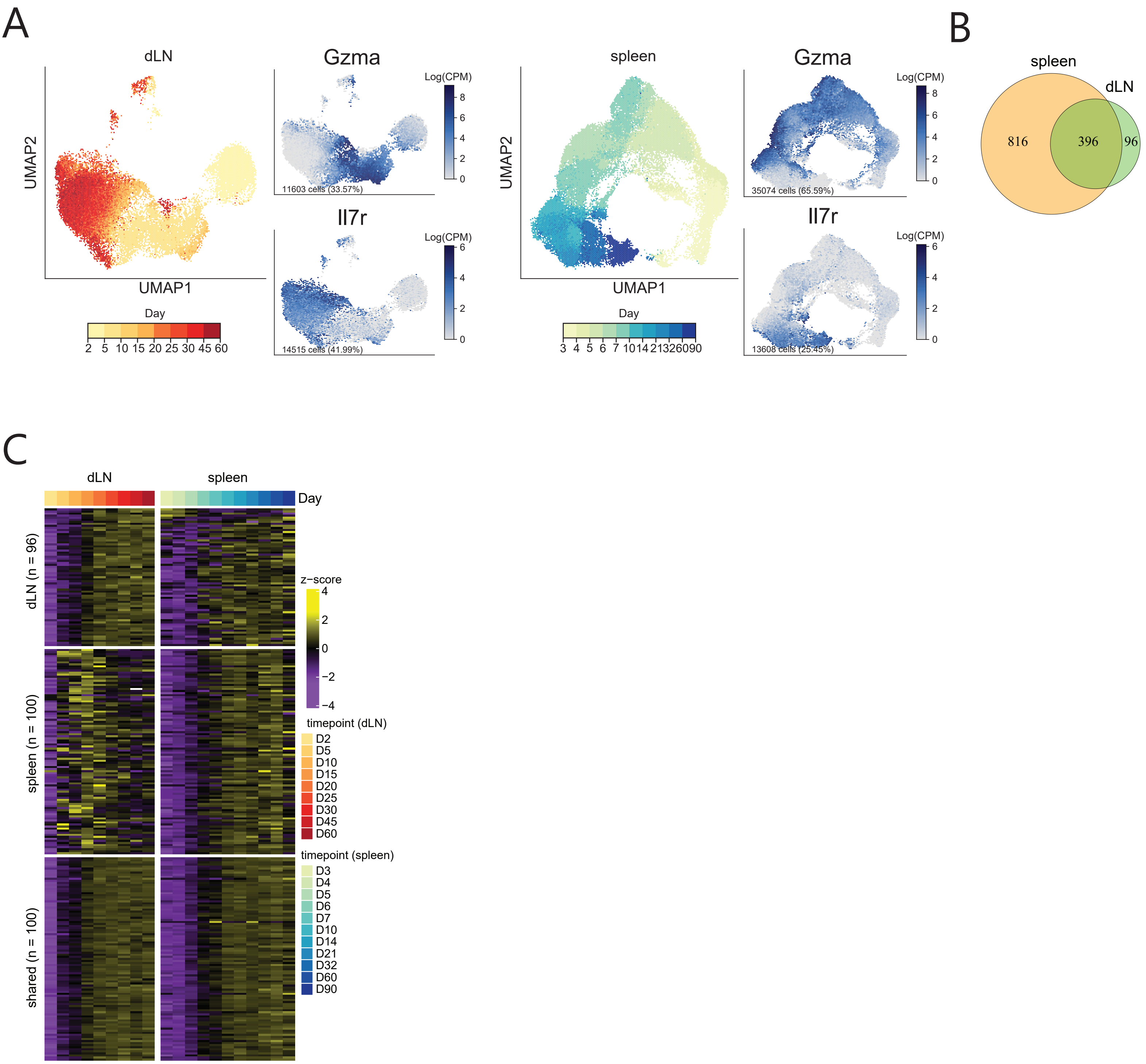

Supplemental Figure 4. Linear modeling reveals temporal circulating T cell gene signature in viral infection models. (A) UMAP embedding of dLN (left) and publicly available spleen data (right) pseudocolored by experimental timepoint. To the right of each timepoint UMAP are feature plots using color to indicate gene expression levels (Log(CPM)) of *Gzma* and *Il7r*. Cell number and percentage represent expression across cells of all timepoints within the given anatomical niche. **(B)** Venn diagram of the significant T_{CIRC} -associated genes in spleen (left) and dLN (right), as determined by linear modeling. **(C)** Heatmap showing the top genes unique to the dLN (top, n = 96 genes),and spleen (middle, n = 100 genes) T_{CIRC} signatures as well as the top genes shared across both anatomical sites (bottom, n = 100). Top bar indicates the associated timepoints. Color scales denote the normalized gene expression (mean zero, unit variance) for each timepoint.

Supplemental Figure 5

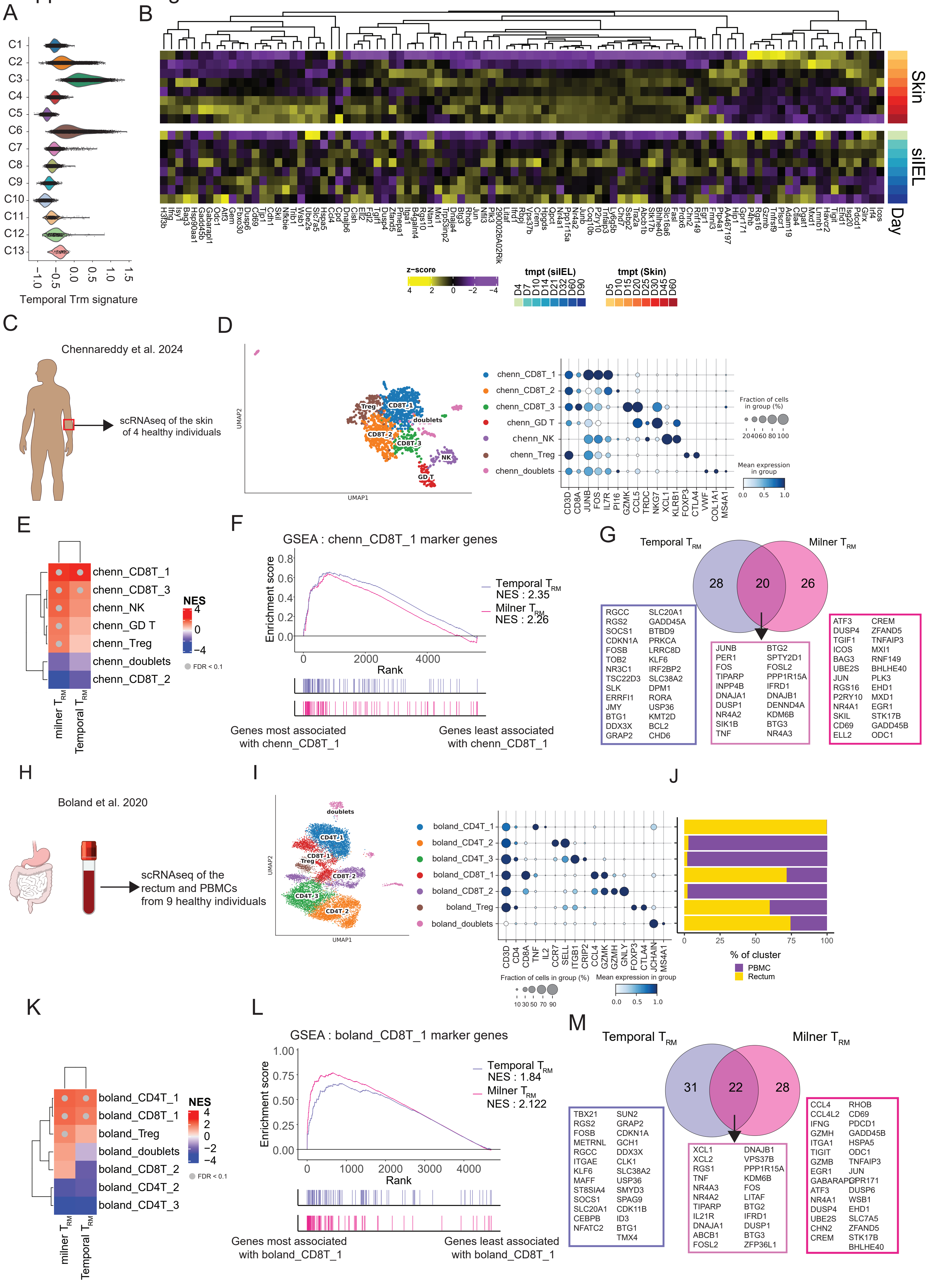

Supplemental Figure 5. T_{RM} signatures in scRNAseq datasets and SCENIC regulon analyses. (A) Gene set scoring of cells using our temporal T_{RM} signature. Violin plots represent the distribution of scores across the cells of each cluster shown in **Figure 1. (B)** Heatmap showing the expression of genes unique to the Milner *et al.*(6) T_{RM} gene signature across timepoints in the skin (left) and siIEL (right) datasets. Color scale denotes normalized gene expression (mean zero, unit variance) for each timepoint. **(C)** Schematic of the dataset used from Chennareddy *et al.* 2024. Only cells from healthy individuals were used for analysis. **(D)** UMAP embedding of 1583 skin T cells colored by annotated cell type (left). (right) Dot plot showing the percentage (size of the dot) and scaled expression (color) of select marker genes for the annotated cell types. **(E)** Heatmap of the normalized enrichment scores when performing GSEA on the subset marker genes of each cell subset with both the Milner *et al.* and the temporal T_{RM} gene sets. Grey dot represents FDR < 0.1. **(F)** GSEA plots using the temporal T_{RM} gene set and the T_{RM} gene set published by Milner *et al.* using subset marker genes as input, where low- and high-ranked genes are most- and least-associated with subset chenn_CD8T_1 respectively. **(G)** Venn diagram of the leading edge genes associated with the GSEA analysis shown in **F**. **(H)** Schematic of the dataset used from Boland *et al.* 2020. Only cells from healthy individuals were used for analysis. **(I)** UMAP embedding of 14053 T cells from the rectum and PBMCs colored by annotated cell type (left). (right) Dot plot showing the percentage (size of the dot) and scaled expression (color) of select marker genes for the annotated cell types. **(J)** Subset composition across tissues. **(K)** Heatmap of the normalized enrichment scores when performing GSEA on the subset marker genes of each cell subset with both the Milner *et al.* and

the temporal T_{RM} gene sets. Grey dot represents FDR < 0.1. **(L)** GSEA plots using the temporal T_{RM} gene set and the T_{RM} gene set published by Milner *et al.* using subset marker genes as input, where low- and high-ranked genes are most- and least-associated with subset boland_CD8T_1 respectively. **(M)** Venn diagram of the leading edge genes associated with the GSEA analysis shown in **L**.

Supplemental Figure 6

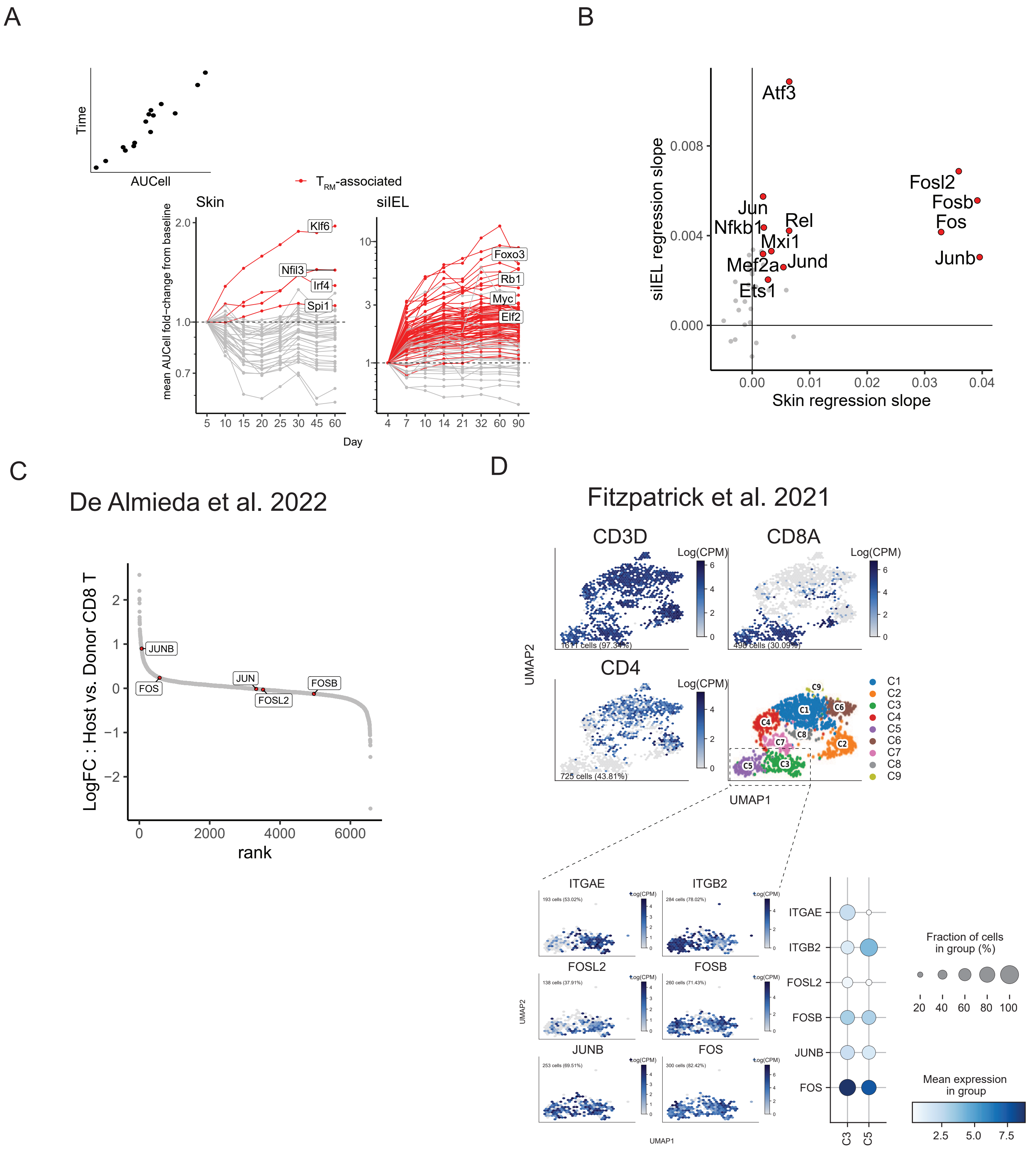

Supplemental Figure 6. AP-1 is associated with T_{RM} in human models. (A) SCENIC regulon analysis of skin (left) and siIEL (right) cells. Scatter plot in the top-left represents the association between time and AUCell scores the analysis is trying to capture. Each line represents a regulon. The x-axis represents the timepoint and the y-axis represents the fold-change of the mean AUCell value of the given regulon at that day when compared to the baseline timepoint (skin = D5, siIEL = D4). Red lines denote significant regulons unique to either skin or siIEL T_{RM} development. **(B)** SCENIC regulon analysis of skin and siIEL cells. Each point represents a regulon and the x- and y- axes represent the regression slope of the mean AUCell score of the regulon in skin and siIEL over time respectively (see **Methods**). Red points denote regulons that were significantly associated with both skin and siIEL T_{RM} development (FDR < 0.1, linear model). **(C)** Gene expression from donor and host CD8 T cells from the skin of a patient 796 days post-allogenic hematopoietic stem cell transplantation were compared. X-axis represents the rank of each gene based on its logFC between host versus donor. AP-1 transcription factor members are labeled(42). **(D)** scRNAseq of donor-derived T cells isolated from an intestinal transplant 1-year post-transplantation(46). (top) UMAP feature plots of canonical T cell markers and clustering of cells. (bottom) UMAP feature plots and dot plot of *ITGAE, ITGB2* and AP-1 transcription factor members. Dot plot shows the percentage (size of the dot) and normalized expression (color) of the genes.

Supplemental methods

Read alignment and quantification

Raw sequencing data was pre-processed with CellRanger (v3.0.2, 10X Genomics) to demultiplex FASTQ reads, align reads to the mouse reference genome (mm10), and count unique molecular identifiers (UMI) to produce a cell x gene count matrix (1). All count matrices were then aggregated with Pegasus (v0.17.2, Python) using the *aggregate matrices* function (2). Droplets with >20% mitochondrial UMI or <500 unique genes detected were deemed low-quality cells or empty droplets and were filtered out of the matrix prior to proceeding with downstream analyses. The counts for each remaining cell in the matrix were then log-normalized by computing the log1p (counts per 100,000), which we refer to in the text and figures as Log(CPM).

Cell clustering

For all cell clustering analysis, 2,000 highly variable genes were selected using the *highly_variable_features* function in Pegasus and used as input for principal component analysis(2). To account for technical variability between batches, the resulting principal component scores were aligned using the Harmony algorithm(3). The top 50 principal components were used as input for generating a neighborhood graph. Clustering the neighborhood graph was performed using the Leiden algorithm(4) and the data was represented using the Uniform Manifold Approximation and Projection (UMAP) algorithm (spread=1, min-dist=0.5)(5) and force-directed layout embedding using the ForceAtlas2 algorithm(6).

Doublet and non-CD8 T cell removal

For all iterations of clustering analysis, cell clusters that were likely to represent cell doublets and/or non-CD8 T cell contaminating populations were filtered using a biologically informed approach. Clusters that had < 60% of cells expressing *Cd3d, Cd8a* and *Trbc2* were removed from the analysis. Additionally, we removed a cluster of cells isolated from lymph nodes that had high expression of both T cell markers (*Cd3d, Cd8a, Trbc2*) and B cell markers (*Ms4a1, Cd79a, Cd19*); these cells were marked as doublets and removed from downstream analyses.

Marker gene identification

The marker genes defining each distinct cell cluster from our skin clustering analysis was determined by applying two complementary methods. First, we captured genes with high expression in each cluster by calculating the area under the receiver operating characteristic (AUROC) curve for the Log(CPM) values of each gene as a predictor of cluster membership using the *de_analysis* function in Pegasus. Genes with an AUROC ≥ 0.75 were considered marker genes for a particular cluster. Second, we created a pseudobulk count matrix to identify genes with lower expression that were highly specific for a given cluster(7). Specifically, we summed the UMI counts across cells for each unique cluster/sample combination to create a matrix of *n genes x (n samples*n clusters)* and performed "one-versus-all" (OVA) differential expression (DE) analyses for each cluster using the *DESeq2* package (v1.32.0, R v4.1.0)(8). For each cluster, we used an input model *gene ~ in_clust*, where *in_clust* is a factor with two levels indicating if the sample was in or not in the cluster being tested. A Wald test was then used to

calculate *P* values and compute a false discovery rate (FDR) using the Benjamini-Hochberg method. We identified marker genes that were significantly associated with a particular cluster as having an FDR<0.05. Marker genes (excluding ribosomal and mitochondrial genes) for each cluster were sequentially identified by first selecting genes with an AUROC ≥ 0.75, followed by those with an OVA pseudobulk FDR <0.05, and up to the top 30 genes were visualized with the *ComplexHeatmap* package (v2.8.0, R)(9). The full list of marker genes is compiled in **Supplemental table 1**.

Pairwise Spearman correlations between clusters

To compare the gene expression profiles of dLN and skin cell subsets, pairwise Spearman correlations between the OVA log2 fold-change values for all genes were calculated using the *cor* function in R. The resulting correlations were visualized with the *ComplexHeatmap* package (v.2.8.0, R).

Waddington-OT trajectory analysis

Waddington-OT was run using the published optimal transport procedure as previously described within the CellRank (v1.5.1, python) suite of tools. Briefly, for a given set of cells C at timepoint T, "descendent" and "ancestor" distributions are estimated as the mass distribution of C_T at later and earlier timepoints respectively. The mass distributions are estimated by transporting C_T to the cells at the neighboring timepoints (C_{T+1}, C_{T-1}), creating "temporal couplings". The temporal couplings for all adjacent timepoints were calculated assuming a uniform growth rate. These temporal couplings were used to examine the relationship between different clusters across timepoints.

Macrostates were calculated using the Generalized Perron Cluster Cluster Analysis (GPCCA) estimator with the *compute_macrostates* function in Cellrank. After defining our mature cell states, absorption probabilities were calculated to estimate the probability of each cell reaching each state using the *compute_absorption_probabilities* function in Cellrank. To define drivers of cell differentiation, the absorption probabilities for each macrostate were correlated with gene expression using the *compute_lineage_drivers* function in Cellrank. The results of these correlations were reduced to only include transcription factors by referencing the mouse TFdb(10).

Analysis of highly variable genes

To look at the pattern of highly variable genes across timecourses, cells from peripheral tissues (i.e., skin, siIEL) were used to create a pseudobulk count matrix where UMI counts were summed across cells for each unique timepoint to create a matrix of *n genes x n timepoints*. The variance of each gene in this matrix was then calculated with the *var* function in R. The top 500 most variable genes were clustered using hierarchical clustering (complete linkage, Euclidean distance) and visualized with the *ComplexHeatmap* package (v2.8.0, R).

Linear modeling of gene expression

To define genes associated with T_{RM} development, cells from peripheral tissues (i.e., skin, siIEL) were used to create a pseudobulk count matrix where UMI counts were summed across cells for each unique timepoint to create a matrix of *n genes x n timepoints*. Linear modeling for each tissue was performed on the pseudobulk count

matrix using the *DESeq2* package (v1.32.0, R v4.1.0). The input model was *gene ~ timepoint* where *timepoint* was a continuous variable with integer values representing the order of timepoints (e.g., in skin : D2 = 0, D5 = 1, D10 = 3). Significant differentially expressed genes (DEG) were defined as those with an FDR < 0.1 (Wald test), were expressed in >5% of cells and had a regression slope > 0.15. Significant genes from these analyses were visualized with *ComplexHeatmap* package (v2.8.0, R). All linear model results can be found in **supplemental table 4**.

SCENIC regulon analysis

To identify putative transcription factors associated with cell development, we employed the SCENIC workflow using Pyscenic (v0.11.2, Python)(11, 12). In short, this workflow first identifies potential transcription factor targets based on their co-expression. These modules of co-expressed genes then undergo cis-regulatory motif enrichment analysis in their corresponding promoters (within 500bp of the transcriptional start site) and are filtered to only include genes that have a corresponding motif. The mm9 motif database provided by the SCENIC authors were used for the motif enrichment analysis. A given transcription factor and filtered target genes are termed a "regulon". The relative activity of these regulons was assessed using the AUCell methodology provided in the SCENIC workflow, where the enrichment of a regulon is calculated relative to non-regulon genes in each cell. To identify regulons associated with temporal development, the mean of the AUCell values for every regulon was taken for the cells from each timepoint to create an AUCell matrix of *n regulons x n timepoints*. We then employed linear modeling on the matrix using the *lm* function in R.

Gene set enrichment analysis

GSEA was performed using the *fgsea* function from the fgsea package (v1.18.0, R) with 10,000 permutations to test for independence. For GSEA performed to find gene sets associated with T_{RM} development, the input gene rankings were based on the linear modeling pseudobulk regression slope values, where the gene with the highest regression slope was ranked first and the lowest regression slope last. For GSEA performed to determine if a given subset was associated with a T_{RM} gene set, the input gene rankings were based on the OVA pseudobulk log-fold-change, where the gene with the highest log-fold-change (I.e. most associated with a cluster) was ranked first and the lowest log-fold-change (i.e. least associated with a cluster) last. For each gene set, an enrichment score that represents the degree a gene set is over-represented at either the highest or lowest end of the regression slope values was calculated and then normalized for differences in gene set sizes.

ATAC-seq and CUT&RUN analysis

Raw sequencing data was first aligned to the mouse reference genome with Bowtie2(13). The resulting BAM files were then filtered to remove mitochondrial reads and PCR duplicates with Picard (v2.27.4, Java). Peak calling was performed for each sample with MACS2 using the BAMPE mode(14). Motif analysis was performed using HOMER software (v4.11.1, Perl) with default settings(15).

1. Zheng GXY, et al. Massively parallel digital transcriptional profiling of single cells. *Nat Commun*. 2017;8:14049.

2. Li B, et al. Cumulus provides cloud-based data analysis for large-scale single-cell and single-nucleus RNA-seq. *Nat Methods*. 2020;17(8):793–798.

3. Korsunsky I, et al. Fast, sensitive and accurate integration of single-cell data with Harmony. *Nat Methods*. 2019;16(12):1289–1296.

4. Traag VA, Waltman L, van Eck NJ. From Louvain to Leiden: guaranteeing well-connected communities. *Sci Rep*. 2019;9(1):5233.

5. McInnes L, Healy J, Melville J. UMAP: Uniform Manifold Approximation and Projection for Dimension Reduction. *arXiv [statML]*. 2018. http://arxiv.org/abs/1802.03426.

6. Jacomy M, et al. ForceAtlas2, a continuous graph layout algorithm for handy network visualization designed for the Gephi software. *PLoS One*. 2014;9(6):e98679.

7. Lun ATL, Bach K, Marioni JC. Pooling across cells to normalize single-cell RNA sequencing data with many zero counts. *Genome Biol*. 2016;17:75.

8. Love MI, Huber W, Anders S. Moderated estimation of fold change and dispersion for RNA-seq data with DESeq2. *Genome Biol*. 2014;15(12):550.

9. Gu Z, Eils R, Schlesner M. Complex heatmaps reveal patterns and correlations in multidimensional genomic data. *Bioinformatics*. 2016;32(18):2847–2849.

10. Kanamori M, et al. A genome-wide and nonredundant mouse transcription factor database. *Biochem Biophys Res Commun*. 2004;322(3):787–793.

11. Aibar S, et al. SCENIC: single-cell regulatory network inference and clustering. *Nat Methods*. 2017;14(11):1083–1086.

12. Van de Sande B, et al. A scalable SCENIC workflow for single-cell gene regulatory network analysis. *Nat Protoc*. 2020;15(7):2247–2276.

13. Langmead B, Salzberg SL. Fast gapped-read alignment with Bowtie 2. *Nat Methods*. 2012;9(4):357–359.

14. Zhang Y, et al. Model-based analysis of ChIP-Seq (MACS). *Genome Biol*. 2008;9(9):R137.

15. Heinz S, et al. Simple combinations of lineage-determining transcription factors prime cis-regulatory elements required for macrophage and B cell identities. *Mol Cell*. 2010;38(4):576–589.

Table legends

Supplemental Table 1. Marker genes for cell subsets. Each row represents a marker gene (gene) for each cellular subset with corresponding AUROC values (AUROC), one-vs.-all (OVA) pseudobulk *P* value (OVA pseudobulk pval), OVA pseudobulk FDR (OVA pseudobulk padj), and OVA pseudobulk log2(fold change) (OVA pseudobulk log-fc), marker gene significance as determined by AUROC (AUROC_marker) and pseudobulk (pseudobulk_marker) approaches and cell subset (subset). Genes with an AUC ≥ 0.75 or a pseudobulk FDR < 0.05 are included for each subset. **Related to Figures 1-2.**

Supplemental Table 2. Waddington-OT transcription factor analysis. Each row represents a transcription factor and included are the statistics looking at the association between the expression of the transcription factors and the C1 and C3 trajectories as determined by Waddington-OT. Statistics included are the correlation associated with each subset (C1_corr, C3_corr), the p-value of the correlation (C1_pval, C3_pval), the FDR of the correlation (C1_qval, C3_qval), the lower limit of the 95% confidence interval (C1_ci_low, C3_ci_low) and the upper limit of the 95% confidence interval (C1_ci_high, C3_ci_high). **Related to figure 2.**

Supplemental Table 3. HOMER motif enrichment results for T_{RM} versus T_{CM}. Each row represents a motif tested for enrichment with HOMER. The columns included are the motif name (Motif Name), the consensus sequence of the motif (Consensus), the P-value for the comparison (P-value), the Log-adjusted p-value (Log P-value), FDR-adjusted p-value (q-value (Benjamini)), the number of target and background

sequences with the given motif (# of Target Sequences with Motif, # of Background Sequences with motif), the percent of target and background sequences with the motif (% of Target Sequences with Motif, % of Background Sequences with Motif) and the comparison being made (contrast), which is either "Trm vs. Tcm" (T_{RM}-associated motis) or "Tcm vs. Trm" (T_{CM}-associated motifs). **Related to figure 2.**

Supplemental Table 4. Linear modeling of genes across time in skin and siIEL. Linear modeling of gene expression across time, where each row represents a gene (gene, gene_ids) and the associated statistics for a given tissue (tissue). The statistics included are the normalized transcript counts averaged for all samples (baseMean), log2 fold-change (log2FoldChange), standard error (lfcSE), Wald-statistic (stat), P-value (pvalue), false discovery rate (padj), the number of cells that had non-zero expression of the gene (n_cells) and the percent of cells that had non-zero expression of the gene (percent_cells). Additionally, it is indicated whether a gene met our statistical criteria as a T_{RM}- or T_{CIRC}- associated gene (met_stat_criteria). **Related to figures 3 and 4.**

Supplemental Table 5. GSEA associated with linear modeling of gene expression over time. Each row represents a gene set (pathway) that was tested for association with the linear modeling results from each tissue (tissue). Included are the associated p-value (pval), false discovery rate (padj), enrichment score (ES), normalized enrichment score (NES), the number of random permutations that had a more extreme enrichment score than the actual data (nMoreExtreme), the number of genes from the

gene set that were found in the linear model results (size) and the leading edge of the GSEA results (leadingEdge). **Related to figure 3.**

Supplemental Table 6. SCENIC linear modeling results. Each row represents a regulon (regulon) and included for each are the linear model statistics for the comparison in a given tissue (tissue). Statistics included are an r-squared value (r_squared), p-value (p_val), regression slope (slope) and false discovery rate (adj_pval). **Related to figures 3 and 4.**

Supplemental Table 7. Software info for computational analyses. Each row represents a package in the coding environment used to generate all figures and included for each is the package version and coding language it corresponds to. **Related to all figures.**
